# Immunogenicity and Safety of COVID-19 Vaccines among People Living with HIV: A Systematic Review and Meta-Analysis

**DOI:** 10.3390/vaccines10091569

**Published:** 2022-09-19

**Authors:** Liangyu Kang, Weijing Shang, Peng Gao, Yaping Wang, Jue Liu, Min Liu

**Affiliations:** School of Public Health, Peking University, Beijing 100191, China

**Keywords:** COVID-19, vaccines, people living with HIV, immunogenicity, safety

## Abstract

Background: The immunogenicity and safety of COVID-19 vaccines among people living with human immunodeficiency virus (PLWH) are unclear. We aimed to evaluate the immunogenicity and safety of COVID-19 vaccines among PLWH. Methods: We systematically searched PubMed, EMBASE, and Web of Science from 1 January 2020 to 28 April 2022 and included observational studies, randomized clinical trials, and non-randomized clinical trials reporting extractable data about the immunogenicity and safety of COVID-19 vaccines among PLWH. Results: A total of 34 eligible studies covering 4517 PLWH were included. The pooled seroconversion rates among PLWH after the first and second doses were 67.51% (95% confident interval (CI) 49.09–85.93%) and 96.65% (95%CI 95.56–97.75%), respectively. The seroconversion was similar between PLWH and healthy controls after the first (risk ratio (RR) = 0.89, 95%CI 0.76–1.04) and the second (RR = 0.97, 95%CI 0.93–1.00) dose. Moreover, the geometric mean titer (GMT) showed no significant difference between PLWH and healthy controls after the first dose (standardized mean difference (SMD) = 0.30, 95%CI -1.11, 1.70) and the second dose (SMD = -0.06, 95%CI -0.18, 0.05). Additionally, the pooled incidence rates of total adverse events among PLWH after the first and the second dose were 46.55% (95%CI 28.29–64.82%) and 30.96% (95%CI 13.23–48.70%), respectively. There was no significant difference in risks of total adverse events between PLWH and healthy controls after the first (RR = 0.86, 95%CI 0.67–1.10) and the second (RR = 0.88, 95%CI 0.68–1.14) dose. Conclusions: The available evidence suggested that the immunogenicity and safety of COVID-19 vaccines among PLWH were acceptable. There was no significant difference in the seroconversion rates and incidence rates of adverse events of COVID-19 vaccines between PLWH and healthy controls.

## 1. Introduction

As a new strain of coronavirus that emerged in 2019, Severe Acute Respiratory Syndrome Coronavirus 2 (SARS-CoV-2) has caused a pandemic of Coronavirus Disease 2019 (COVID-19) around the world. By 23 May 2022, COVID-19 has caused more than 500 million cumulative confirmed cases and 6.28 million cumulative deaths worldwide [1]. It has posed a great challenge to healthcare systems and will continue to be a threat to global health [2,3]. SARS-CoV-2 is a highly transmissible and pathogenic coronavirus that can be transmitted through various routes including air and direct and indirect contact [4]. Noteworthily, compared with the general population, people with other underlying diseases or immunocompromised individuals display greater morbidity and mortality from COVID-19 [5,6].

People living with human immunodeficiency virus (PLWH) might be more susceptible to SARS-CoV-2 infection and more likely to present with severe COVID-19 when infected due to lower immune responses and viral interactions [7,8]. According to a report from World Health Organization (WHO), human immunodeficiency virus (HIV) infection appears to be a significant independent risk factor for acquiring SARS-CoV-2 infection and is associated with a higher risk of mortality from COVID-19 [7]. Currently, specific medicine to treat COVID-19 has not yet been developed [9], whereas available evidence shows that public health control measures and vaccination are effective measures in reducing morbidity and mortality from the disease [10]. Among all the measures, vaccination is considered to be the most cost-effective and efficient way [11]. As of 23 May 2022, more than 11.8 billion COVID-19 vaccines have been administered globally [1]. The immunogenicity and safety of vaccines are very important to protect people from infection, particularly for PLWH. Although many studies reported data from the general population [12,13,14], the immunogenicity and safety of vaccination remain unclear in PLWH, which hinders their willingness to actively get vaccinated [15,16,17].

Studies on the immunogenicity and safety of COVID-19 vaccines among PLWH have been conducted in different countries, but the conclusions are still contradictory. For immunogenicity, some studies showed that protective antibody responses in PLWH were inferior to those in healthy individuals [18,19], while the levels of protective antibodies were similar between the two populations in some randomized clinical trials (RCTs) [20,21,22]. For safety, some studies found higher incidence rates of adverse events in PLWH [18,23], whereas other studies indicated that the incidence rates of adverse events in PLWH were not different from or even lower than that in the general population [18,22,23,24]. Therefore, this meta-analysis aimed to systematically evaluate the immunogenicity and safety of COVID-19 among PLWH by reviewing the published relevant studies, thereby providing evidence-based references for PLWH in regard to COVID-19 vaccines.

## 2. Materials and Methods

### 2.1. Search Strategy

We conducted the meta-analysis following the Preferred Reporting Items for Systematic Reviews and Meta-Analyses (PRISMA) guideline [25]. This review was registered with PROSPERO (CRD42022329167). Two researchers (L.K. and W.S.) searched the published studies between 1 January 2020 and 28 April 2022, through PubMed, EMBASE, and Web of Science with English-language restrictions. The search terms included (“SARS-CoV-2” or “COVID-19”) and (“HIV” or “acquired immunodeficiency syndrome”) and (“COVID-19 Vaccines” or “Vaccines” or “Vaccination”). The detailed search strategies are shown in Text S1 in the Supplemental Materials. Two researchers (L.K. and W.S.) reviewed the titles, abstracts, and full texts of articles independently and identified additional studies from the reference lists. Disagreements were resolved by two other reviewers (P.G. and Y.W.).

The primary outcome to evaluate the immunogenicity of COVID-19 vaccines was the seroconversion of neutralizing antibodies to SARS-CoV-2 after a first or second dose, defined as a change from seronegative at baseline to seropositive [26]. The calculation formula was seroconversion rate = the number of people with seroconversion/number of people receiving COVID-19 vaccines × 100%. The geometric mean titer (GMT) of neutralizing antibodies was also used to assess the immunogenicity. The safety of COVID-19 vaccines was determined in this study as the incidence rate of adverse events after vaccination including systemic and local adverse events [27]. The calculation formula was: incidence rate of adverse events = number of people having adverse events/number of people receiving COVID-19 vaccines × 100%.

### 2.2. Inclusion and Exclusion Criteria

The inclusion criteria consist of (1) studies reporting PLWH receiving any COVID-19 vaccines who had never been infected with SARS-CoV-2; (2) observational studies (cross-sectional studies, case-control studies, and cohort studies), non-randomized clinical trials, and RCTs; (3) studies with extractable data on seroconversion rates, GMT, and incidence rates of adverse events. We excluded the following studies: (1) non-original articles such as reviews, comments, letters, etc.; (2) articles unable to find full text; (3) preprints; (4) studies with insufficient data to calculate the seroconversion rate and incidence rate of adverse events.

### 2.3. Data Extraction

The following data were extracted independently by two researchers (L.K. and W.S.) from the included studies: (1) basic information of the studies, including first author, publication year, country, and study design; (2) characteristics of the study population, including the number of PLWH receiving COVID-19 vaccines, and CD4+ T cell counts; (3) relevant information on vaccines, involving types of COVID-19 vaccines, dose, and the time interval between vaccination and antibody testing; (4) outcome for the immunogenicity, including the number of PLWH with seroconversion and GMT of neutralizing antibodies; (5) outcome for the safety, involving the number of PLWH having adverse events. If available, we also collected the data on the immunogenicity and safety of COVID-19 vaccines among healthy controls in cohort studies, non-randomized clinical trials, and RCTs, including the number of healthy controls receiving COVID-19 vaccines, number of healthy controls with seroconversion, and number of healthy controls having adverse events.

### 2.4. Risk of Bias Assessment

We evaluated the risk of bias using the Revised Cochrane risk-of-bias tool for randomized trials (RoB 2) [28] for RCTs, Risk Of Bias In Non-randomized Studies of Interventions (ROBINS-I) tool [29] for non-randomized clinical trials, Newcastle–Ottawa scale [30] for cohort studies and case-control studies, and Agency for Healthcare Research and Quality (AHRQ) [31] for cross-sectional studies. Two researchers (L.K. and W.S.) performed the quality assessment independently. Disagreements were resolved by two other reviewers (P.G. and Y.W.).

### 2.5. Data Synthesis and Statistical Analysis

Based on available data about seroconversion rates and incidence rates of adverse events from observational studies, non-randomized clinical trials, and RCTs, we estimated the pooled seroconversion rates and incidence rates of adverse events as well as their 95% confidence intervals (CIs) among PLWH receiving a first or second dose of COVID-19 vaccines, using the inverse variance-weighted random-effects model [32].

For cohort studies, non-randomized clinical trials, and RCTs with healthy controls, the crude risk ratios (RRs) of seroconversion and adverse events were calculated using the following formula.

(1) $$RR=\frac{n_{p}\;/\;N_{p}}{n_{c}\;/\;N_{c}}$$

The *n_p_* represented the number of PLWH with seroconversion or having adverse events; *N_p_* represented the number of PLWH receiving COVID-19 vaccines; *n_c_* represented the number of healthy controls with seroconversion or having adverse events; *N_c_* represented the number of healthy controls receiving COVID-19 vaccines. The Mantel-Haenszel random-effects method [33] was adopted to calculate the pooled RRs and their 95%CIs, to compare the seroconversion and safety between PLWH and a healthy population. Both RR and the lower limit of its 95%CI > 1 indicated that PLWH had a higher risk of seroconversion and adverse events after vaccination compared with healthy controls; both RR and the upper limit of its 95%CI < 1 indicated that PLWH had a lower risk; other situations suggested no significant difference between PLWH and healthy controls.

For observational studies and trials with data about GMT of neutralizing antibodies, standardized mean difference (SMD) was used to compare GMT among PLWH with healthy controls. The heterogeneity among studies was assessed using *I^2^* values, and *I^2^* ≥ 50% was regarded as significant heterogeneity [34].

We conducted subgroup analyses by continent, study design, vaccine type, time interval between vaccination and antibody testing, and CD4+ T cell counts. We used the *Q* test to conduct subgroup comparisons and variables were considered significant between subgroups if the subgroup difference *p* value was less than 0.05. The studies with a high risk of bias were excluded for sensitivity analysis. We also performed sensitivity analysis by excluding studies with a number of PLWH < 100 as studies with small sizes are susceptible to selection bias and tend to have larger treatment effects than large studies [35]. Publication bias was assessed by funnel plot and Egger’s regression test. When publication bias was suspected based on either the funnel plot or Egger’s test, we conducted a sensitivity analysis using the trim-and-fill method to re-estimate the pooled effect size after imputing potentially missing studies [36]. Two-sided *p* < 0.05 indicated statistical significance. All analyses were performed on R (version 4.0.5) using the *meta* and *forestplot* packages.

## 3. Results

### 3.1. Characteristics of Included Studies

We identified 4250 studies through databases search and reference lists of articles and reviews. 1088 duplicates were excluded. After reading titles and abstracts, we excluded 3042 irrelevant articles. Among the 120 studies under full-text review, 86 studies were excluded. The final meta-analysis comprised 34 eligible studies, including 22 articles [21,22,23,37,38,39,40,41,42,43,44,45,46,47,48,49,50,51,52,53,54,55] for only immunogenicity, three articles [24,56,57] for only safety, and nine articles [18,20,58,59,60,61,62,63,64] for both immunogenicity and safety (Figure S1).

Among the 34 studies, seven (20.59%) were cross-sectional studies, one (2.94%) was a case-control study, 18 (52.94%) were cohort studies, six (17.65%) were non-randomized clinical trials, two (5.88%) were RCTs. 13 (38.24%) studies were conducted in Europe, 10 (29.41%) in Asia, seven (20.59%) in North America, two (5.88%) in South America, and two (5.88%) in Africa. 13 (38.24%) studies were assessed as low risk of bias, 17 (50.00%) as the moderate risk of bias, and four (11.76%) as high risk of bias. Characteristics of included studies are shown in Tables S1–S2, and results of risk of bias assessment are detailed in Tables S3–S7.

### 3.2. Seroconversion Rates among PLWH

**Figure 1:**
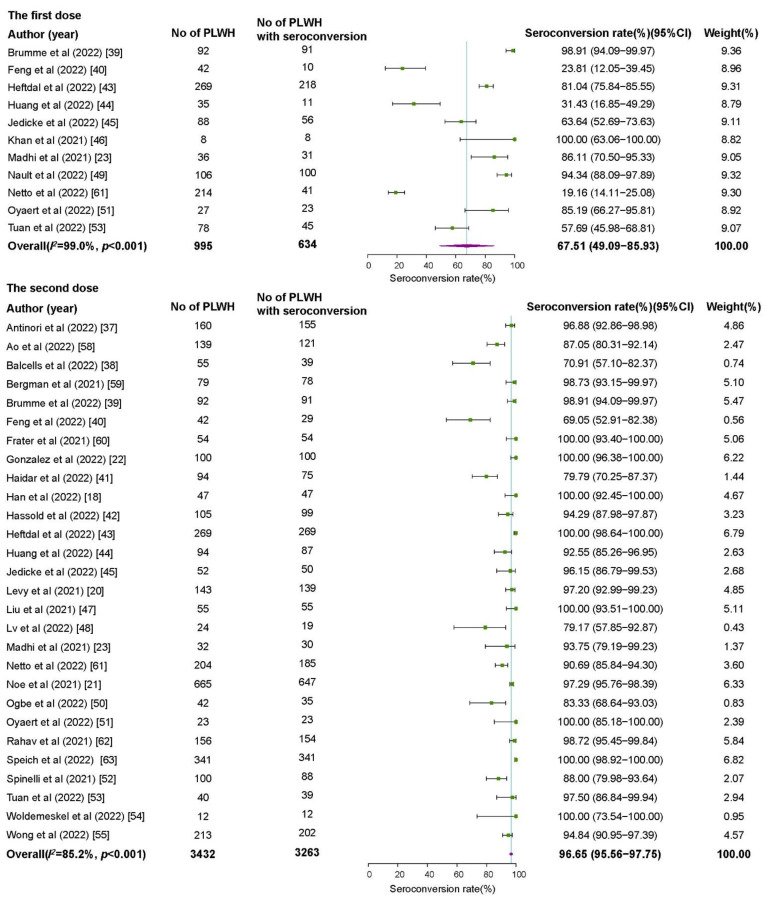
The seroconversion rates of SARS-CoV-2 antibodies among people living with HIV. PLWH: people living with HIV; CI: confidence interval.

In 11 studies involving 995 PLWH receiving the first dose of COVID-19 vaccines, the pooled seroconversion rate was 67.51% (95%CI 49.09–85.93%), with high heterogeneity among studies (*I*^2^ = 99.0%) (Figure 1). The subgroup analyses showed significant differences in seroconversion rates among different continents, study designs, and vaccine types (*p* < 0.05). The seroconversion rates were relatively lower in South America (19.16%, 95%CI 13.89–24.43%), cross-sectional studies (45.14%, 95%CI 19.42–70.85%), and PLWH receiving inactivated virus vaccines (21.69%, 95%CI 15.74–27.63%) (Table 1).

In 28 studies involving 3432 PLWH receiving the second dose of COVID-19 vaccines, the pooled seroconversion rate was 96.65% (95%CI 95.56–97.75%), with high heterogeneity among studies (*I*^2^ = 85.2%) (Figure 1). Significant subgroup differences were observed in different continents, study designs, vaccine types, time intervals between vaccination and antibody testing, and CD4+ T cell counts (*p* < 0.05). The PLWH in South America (81.64%, 95%CI 62.33–100.00%), receiving inactivated virus vaccines (88.62%, 95%CI 83.21–94.03%), whose time intervals between vaccination and antibody testing > 28 days (92.89%, 95%CI 89.40–96.38%), and with CD4+ T cell counts < 500 cells/μL (91.44%, 95%CI 85.77–97.11%) had lower seroconversion rates (Table 1).

### 3.3. Comparison of Seroconversion between PLWH and Healthy Controls

**Figure 2:**
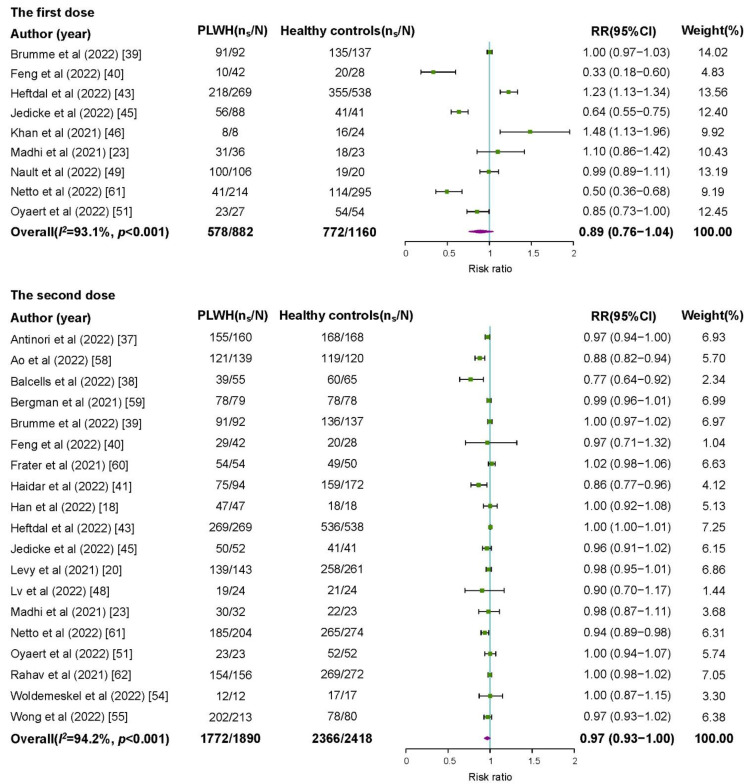
Risk ratios for seroconversion among people living with HIV compared with healthy controls after a first or second dose of COVID-19 vaccine. PLWH: people living with HIV; n_s_: number of people with seroconversion; N: group size; RR: risk ratio; CI: confidence interval.

In nine studies consisting of 882 PLWH and 1160 healthy controls after the first dose of COVID-19 vaccines, the risk of achieving seroconversion was not significantly different between PLWH and healthy controls (RR = 0.89, 95%CI 0.76–1.04), with high heterogeneity among studies (*I*^2^ = 93.1%) (Figure 2). The subgroup analyses showed significant differences in RRs among different continents and vaccine types (*p* < 0.05). The risk of seroconversion among PLWH in Asia (RR = 0.33, 95%CI 0.18–0.60), in South America (RR = 0.50, 95%CI 0.36–0.68), and receiving inactivated virus vaccines (RR = 0.44, 95%CI 0.31–0.63) was lower than that among healthy controls (Table 2).

In 19 studies consisting of 1890 PLWH and 2418 healthy controls after the second dose of COVID-19 vaccines, the risk of seroconversion was similar between PLWH and healthy controls (RR = 0.97, 95%CI 0.93–1.00), with high heterogeneity among studies (*I*^2^ = 94.2%) (Figure 2). The risk of seroconversion among PLWH was not significantly different from healthy controls in each subgroup (RRs’ 95%CIs cross 1), except for PLWH who received inactivated virus vaccines (RR = 0.92, 95%CI 0.87–0.97) (Table 2).

### 3.4. Geometric Mean Titers between PLWH and Healthy Controls

In two studies involving 137 PLWH and 73 healthy controls after the first dose of COVID-19 vaccines, the GMT showed a nonsignificant difference between the two groups (SMD = 0.30, 95%CI −1.11, 1.70). In five studies consisting of 571 PLWH and 681 healthy controls after the second dose of COVID-19 vaccines, the GMT among PLWH was not significantly different from that among healthy controls (SMD = −0.06, 95%CI −0.18, 0.05) (Figure S2).

### 3.5. Safety of COVID-19 Vaccines among PLWH

**Figure 3:**
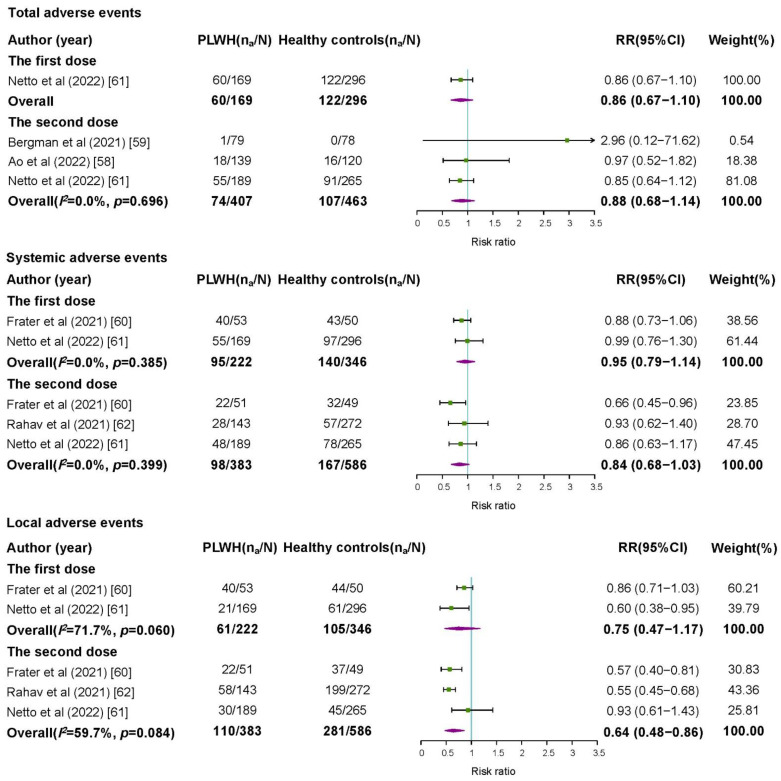
Risk ratios for adverse events among people living with HIV compared with healthy controls after a first or second dose of COVID-19 vaccine. PLWH: people living with HIV; n_a_: number of people reported adverse events; N: group size; RR: risk ratio; CI: confidence interval.

After the first dose of COVID-19 vaccines, the pooled incidence rates of total adverse events, systemic adverse events, and local adverse events were 46.55% (95%CI 28.29–64.82%), 39.48% (95%CI 17.58–61.38%), and 42.94% (95%CI 21.14–64.74%), respectively (Figure S3). There was no significant difference in risks of total adverse events (RR = 0.86, 95%CI 0.67–1.10), systemic adverse events (RR = 0.95, 95%CI 0.79–1.14), and local adverse events (RR = 0.75, 95%CI 0.47–1.17) between PLWH and healthy controls (Figure 3).

After the second dose of COVID-19 vaccines, the pooled incidence rates of total adverse events, systemic adverse events, and local adverse events were 30.96% (95%CI 13.23–48.70%), 33.75% (95%CI 22.90–44.60%), and 36.98% (95%CI 19.83–54.13%), respectively (Figure S3). The risks of total adverse events (RR = 0.88, 95%CI 0.68–1.14) and systemic adverse events (RR = 0.84, 95%CI 0.68–1.03) in PLWH were compatible with those in healthy controls, and the risk of local adverse events was even slightly lower in PLWH (Figure 3).

### 3.6. Sensitivity Analysis and Publication Bias

After excluding four studies with a high risk of bias, the pooled seroconversion rates, RRs for seroconversion, and incidence rates of adverse events were close to the original results (Figures S4–S6). After excluding studies with the number of PLWH < 100, the results also remained stable (Figures S7–S9). The funnel plots and Egger’s test suggested that there might be publication bias in the meta-analyses of seroconversion rates and RRs for seroconversion after the second dose of COVID-19 vaccines (Figures S10–S12). Using the trim-and-fill method to address publication bias, the pooled seroconversion rate (99.23%, 95%CI 98.03–100.00%) and RR (1.00, 95%CI 0.98–1.02) were very close to the original results.

## 4. Discussion

In this systematic review and meta-analysis, we found that the pooled seroconversion rate among PLWH after the second dose (96.65%) was higher than that after the first dose (67.51%). Subgroup analyses showed that PLWH receiving inactivated virus vaccines had lower seroconversion rates after both doses, and lower seroconversion rates were observed among PLWH whose time intervals between vaccination and antibody testing > 28 days and CD4+ T cell counts < 500 cells/μL. Compared with healthy controls, the risk of seroconversion among PLWH receiving inactivated virus vaccines was lower (RR = 0.92, 95%CI 0.87–0.97) than that among healthy controls. Moreover, the GMT showed no significant difference between PLWH and healthy controls after the first dose and the second dose. In addition, we also found there was no significant difference in the safety of COVID-19 vaccines between PLWH and health controls. The pooled incidence rates of total adverse events after the first dose and after the second dose were 46.55% and 30.96%, respectively. PLWH even had a slightly lower risk of local adverse events than healthy controls (RR = 0.64, 95%CI 0.48–0.86).

Our results suggested that the seroconversion of COVID-19 vaccines was compatible between PLWH and healthy individuals, and the pooled seroconversion rate after the second dose was higher than that after the first dose among PLWH. Nowadays, there are very few relevant systematic reviews. Lee et al. [65] conducted a systematic review on the efficacy of COVID-19 vaccines in immunocompromised patients and found the seroconversion in PLWH was similar to the immunocompetent population after the second dose (RR = 1.00, 95%CI 0.98–1.01). Our results were consistent with Lee’s study. Furthermore, our study gave a more comprehensive picture of the immunogenicity in PLWH by including more studies and a larger PLWH population. Our findings highlighted the importance of receiving a second dose of the COVID-19 vaccine in PLWH.

In the subgroup analyses, we found that PLWH receiving inactivated virus vaccines had lower seroconversion rates after both doses, and their risk of seroconversion was lower than healthy controls. Currently, there has been no systematic review reporting the immunogenicity of different types of COVID-19 vaccines among PLWH. Cheng et al. [66] evaluated the effectiveness and safety of different types of COVID-19 vaccines through a systematic review of the general population and found that all the vaccines had excellent effectiveness and acceptable risk of adverse events. Among various types of COVID-19 vaccines, the inactivated vaccine had lower effectiveness but higher safety. Additionally, lower seroconversion rates were observed among PLWH whose time intervals between vaccination and antibody testing > 28 days and CD4+ T cell counts < 500 cells/μL. Several observational studies and non-randomized clinical trials [18,40,58] also showed that the concentration of protective antibodies decreased significantly in PLWH after vaccination than that in healthy individuals. Therefore, booster vaccination might be important to prevent primary and re-infection of SARS-CoV-2 in PLWH. The immune system of PLWH is weakened due to the declined number of CD4+ T cells. Correspondingly, their impaired cellular and humoral immunity might limit the immune responses elicited by vaccines [67]. Netto et al. [61] conducted a prospective cohort study covering 215 PLWH and found that PLWH whose CD4+ T cell counts were less than 500 cells/μL had lower seroconversion rates than those with CD4+ T cell counts of at least 500 cells/μL. These findings indicated strategies should be developed to improve vaccine-induced immunogenicity in PLWH, especially in the subgroup with lower CD4+ T cell counts. Furthermore, the immune-related functions and HIV viral load in PLWH should be monitored carefully before and after vaccination.

In this study, we also found that there was no significant difference in the safety of COVID-19 vaccines between PLWH and health controls. The pooled incidence rates of total adverse events after the first dose and the second dose were 46.55% and 30.96%, respectively. The risk of local adverse events was even slightly lower in PLWH (RR = 0.64, 95%CI 0.48–0.86) compared with healthy controls. The pooled incidence rates of adverse events in PLWH were close to results from previous studies in healthy populations. A meta-analysis including 12 clinical trials covering 22802 vaccine recipients indicated that 46.3% (95%CI 38.2–54.3%) of them reported at least one systemic adverse event and 66.7% (95%CI 53.2–80.3%) reported at least one local adverse event after the first dose [27]. Moreover, we found a lower incidence rate of adverse events among PLWH after the second dose in comparison to that after the first dose, consistent with several published studies [12,57,61]. The reason might be that people having fewer adverse events after the first dose were more likely to receive a second dose. Nevertheless, our study demonstrated the safety of COVID-19 vaccines and would be helpful to mitigate vaccine hesitancy and concerns in PLWH.

To our knowledge, this is the first study to systematically evaluate the immunogenicity and safety of the COVID-19 vaccine in PLWH. Relevant studies on PLWH receiving a first or second dose of the COVID-19 vaccine published from 1 January 2020 to 29 April 2022 were included. We estimated the pooled seroconversion rates of protective antibodies and incidence rates of adverse events among PLWH and performed subgroup analyses among different continents, study designs, vaccine types, and CD4+ T cell counts. We also found that the immunogenicity and safety of COVID-19 vaccines were similar between PLWH and healthy controls. Our results could help reduce vaccine hesitancy and concerns among PLWH and provide evidence-based references for policymakers to make vaccination strategies.

This study has several limitations. First, we did not evaluate the immunogenicity and safety of booster doses of COVID-19 vaccines in PLWH due to lacking original studies. More studies on booster vaccination among PLWH are needed. Second, the majority of the included studies were conducted in Europe and Asia, while there were limited studies in Africa where the disease burden of HIV is heavy. Therefore, our results should be interpreted with caution when applying to PLWH in Africa. In the future, relevant studies in Africa are required to further complement the immunogenicity and safety of COVID-19 vaccines among PLWH. Third, the high heterogeneity among studies which might be related to different study locations, periods, and sample sizes, made the results in need of future verification.

## 5. Conclusions

In conclusion, the available evidence suggested that the immunogenicity and safety of COVID-19 vaccines among PLWH were acceptable. There was no significant difference in the seroconversion rates and incidence rates of adverse events of COVID-19 vaccines between PLWH and healthy controls. Further studies on the immunogenicity, effectiveness and safety of COVID-19 vaccines should focus on various types of vaccines, PLWH with different CD4+ T cell counts, and booster vaccination, especially in countries and regions with heavy HIV burdens.

## Figures and Tables

**Table 1:** The seroconversion rates of SARS-CoV-2 antibodies among people living with HIV by subgroup.

	No. of Studies	No. of PLWH	Seroconversion Rate (%) (95%CI)	*I*^2^ (%)	*p* Value for Heterogeneity	Weight (%)	*p* Value for Subgroup Differences
**The first dose**							
**Overall**	11	995	67.51 (49.09–85.93)	99.0	<0.001	100.0	
**Continent**							<0.001
Africa	2	44	92.15 (78.66–100.00)	52.6	0.147	17.9	
Asia	2	77	26.95 (17.08–36.83)	0.0	0.457	17.7	
Europe	3	283	76.51 (64.51–88.51)	80.9	0.005	27.3	
North America	3	276	85.19 (78.66–100.00)	96.3	<0.001	27.7	
South America	1	214	19.16 (13.89–24.43)	NA	NA	9.3	
**Study design**							0.036
Cross-sectional study	2	113	45.14 (19.42–70.85)	86.5	0.006	17.9	
Cohort study	6	796	73.71 (49.11–98.30)	99.4	<0.001	55.3	
Non-randomized clinical trial	2	50	61.81 (0.00–100.00)	98.3	<0.001	17.8	
Randomized clinical trial	1	36	86.11 (74.81–97.41)	NA	NA	9.0	
**Vaccine type**							<0.001
Adenovirus vector vaccines	2	44	92.15 (78.66–100.00)	52.6	0.147	19.7	
Inactivated virus vaccines	3	291	21.69 (15.74–27.63)	17.2	0.299	29.9	
mRNA vaccines	5	568	76.81 (64.23–89.38)	93.4	<0.001	50.4	
**Time interval between** **vaccination and antibody testing**							0.623
< 28 days	5	568	76.81 (64.23–89.38)	93.4	<0.001	50.1	
≥ 28 days	5	392	65.55 (22.55–100.00)	99.5	<0.001	49.9	
**CD4+ T-cell counts**							0.369
<500 cells/μL	1	64	15.62 (6.73–24.52)	NA	NA	NA	
≥500 cells/μL	1	150	20.67 (14.19–27.15)	NA	NA	NA	
**The second dose**							
**Overall**	28	3432	96.65 (95.56–97.75)	85.2	<0.001	100.0	
**Continent**							0.011
Africa	1	32	93.75 (85.36–100.00)	NA	NA	1.4	
Asia	9	913	94.83 (91.82–97.84)	83.8	<0.001	31.1	
Europe	11	1890	98.87 (97.96–99.77)	74.1	0.007	50.3	
North America	5	338	93.11 (86.66–99.56)	85.9	<0.001	12.9	
South America	2	259	81.64 (62.33–100.00)	89.4	0.002	4.3	
**Study design**							0.014
Cross-sectional study	6	1059	97.74 (95.80–99.67)	72.3	0.003	26.4	
Cohort study	14	1659	95.55 (93.45–97.65)	87.4	<0.001	51.3	
Case-control study	1	100	88.00 (81.63–94.37)	NA	NA	2.1	
Non-randomized clinical trial	5	241	90.53 (83.83–97.21)	87.0	<0.001	12.0	
Randomized clinical trial	2	373	98.34 (92.92–100.00)	53.0	0.145	8.2	
**Vaccine type**							<0.001
Adenovirus vector vaccines	3	128	93.68 (84.63–100.00)	79.0	0.009	8.2	
Inactivated virus vaccines	9	734	88.62 (83.21–94.03)	89.6	<0.001	24.2	
mRNA vaccines	13	1614	99.14 (98.43–99.85)	57.6	0.005	67.6	
**Time interval between** **vaccination and antibody testing**							0.011
≤14 days	8	653	98.66 (96.78–100.00)	73.7	<0.001	38.8	
15–28 days	6	620	95.34 (92.04–98.83)	87.1	<0.001	27.8	
>28 days	9	1070	92.89 (89.40–96.38)	89.7	<0.001	33.3	
**CD4+ T-cell counts**							0.044
<500 cells/μL	4	224	91.44 (85.77–97.11)	58.6	0.064	42.6	
≥500 cells/μL	3	270	97.99 (95.09–100.00)	65.5	0.055	57.4	

PLWH: people living with HIV; CI: confidence interval.

**Table 2:** Risk ratios for seroconversion among full-vaccinated people living with HIV compared with healthy controls by subgroup.

	No. of Studies	No. of PLWH	RR (95%CI)	*I*^2^ (%)	*p* Value for Heterogeneity	Weight (%)	*p* Value for Subgroup Differences
**The first dose**							
**Overall**	9	882	0.89 (0.76–1.04)	93.1	<0.001	100.0	
**Continent**							<0.001
Africa	2	44	1.27 (0.93–1.74)	64.3	0.094	20.4	
Asia	1	42	0.33 (0.18–0.60)	NA	NA	4.8	
Europe	3	384	0.88 (0.58–1.33)	96.8	<0.001	38.4	
North America	2	198	1.00 (0.97–1.03)	0.0	0.827	27.2	
South America	1	214	0.50 (0.36–0.68)	NA	NA	9.2	
**Study design**							0.291
Cohort study	6	796	0.87 (0.73–1.02)	94.2	<0.001	74.8	
Non-randomized clinical trial	2	50	0.71 (0.09–5.87)	97.6	<0.001	14.8	
Randomized clinical trial	1	36	1.10 (0.86–1.42)	NA	NA	10.4	
Vaccine type							<0.001
Adenovirus vector vaccines	2	44	1.27 (0.93–1.74)	64.3	0.094	24.7	
Inactivated virus vaccines	2	256	0.44 (0.31–0.63)	27.6	0.240	19.3	
mRNA vaccines	4	490	0.91 (0.69–1.20)	95.2	<0.001	56.0	
**Time interval between** **vaccination and antibody testing**							0.753
<28 days	4	490	0.91 (0.69–1.20)	95.2	<0.001	51.6	
≥28 days	5	392	0.78 (0.31–1.94)	98.8	<0.001	48.4	
**CD4+ T-cell count**							0.421
<500 cells/μL	1	64	0.40 (0.22–0.73)	NA	NA	25.7	
≥500 cells/μL	1	150	0.53 (0.38–0.76)	NA	NA	74.3	
**The second dose**							
**Overall**	19	1890	0.97 (0.93–1.00)	94.2	<0.001	100.0	
**Continent**							0.643
Africa	1	32	0.98 (0.87–1.11)	NA	NA	3.7	
Asia	7	764	0.97 (0.93–1.01)	69.8	0.003	33.5	
Europe	6	637	0.99 (0.97–1.02)	81.6	<0.001	39.7	
North America	3	198	0.95 (0.78–1.16)	92.9	<0.001	14.4	
South America	2	259	0.86 (0.70–1.07)	80.5	0.024	8.7	
**Study design**							0.342
Cohort study	14	1659	0.96 (0.92–1.00)	96.1	<0.001	80.2	
Non-randomized clinical trial	4	199	1.00 (0.97–1.03)	17.9	0.301	16.1	
Randomized clinical trial	1	32	0.98 (0.87–1.11)	NA	NA	3.7	
**Vaccine type**							0.013
Adenovirus vector vaccines	2	86	1.02 (0.98–1.06)	0.0	0.444	10.8	
Inactivated virus vaccines	7	585	0.92 (0.87–0.97)	47.7	0.075	25.4	
mRNA vaccines	9	1033	0.99 (0.97–1.00)	72.9	<0.001	63.8	
**Time interval between** **vaccination and antibody testing**							0.689
≤14 days	7	598	0.98 (0.93–1.04)	43.8	<0.001	43.8	
15–28 days	4	480	0.96 (0.90–1.02)	84.9	<0.001	22.8	
>28 days	6	587	0.95 (0.91–1.00)	81.9	<0.001	33.4	
**CD4+ T-cell count**							0.178
<500 cells/μL	3	170	0.93 (0.86–1.01)	69.0	0.040	48.9	
≥500 cells/μL	2	219	0.98 (0.96–1.01)	0.0	0.501	51.1	

PLWH: people living with HIV; RR: risk ratio; CI: confidence interval.

## Data Availability

Data can be obtained by contacting the corresponding author.

## Supplementary Materials

The following supporting information can be downloaded at: https://www.mdpi.com/article/10.3390/vaccines10091569/s1, Text S1: Detailed Search Strategies; Figure S1: Flowchart of study selection; Figure S2: The SMD of geometric mean titer among people living with HIV and healthy controls.; Figure S3: The incidence rates of adverse events among people living with HIV.; Figure S4: Sensitivity analysis of seroconversion rates by excluding studies with a high risk of bias.; Figure S5: Sensitivity analysis of risk ratio of seroconversion by excluding studies with a high risk of bias.; Figure S6: Sensitivity analysis of incidence rates of adverse events by excluding studies with a high risk of bias.; Figure S7: Sensitivity analysis of seroconversion rates by excluding studies with the number of people living with HIV less than 100; Figure S8: Sensitivity analysis of risk ratio of seroconversion by excluding studies with the number of people living with HIV less than 100; Figure S9 Sensitivity analysis of incidence rates of adverse events by excluding studies with the number of people living with HIV less than 100; Figure S10: The publication bias of studies on seroconversion rates among people living with HIV after a first or second dose of COVID-19 vaccine; Figure S11: The publication bias of studies on seroconversion among people living with HIV compared with healthy controls after a first or second dose of COVID-19 vaccine; Figure S12: The publication bias of studies on incidence rates of adverse events among people living with HIV after a first or second dose of COVID-19 vaccine; Table S1: Characteristics and basic information of the studies included in the systematic review and meta-analysis for COVID-19 vaccine immunogenicity; Table S2: Characteristics and basic information of the studies included in the systematic review and meta-analysis for COVID-19 vaccine safety; Table S3: Risk of bias of all included randomized clinical trials using the revised Cochrane risk-of-bias tool for randomized trials (RoB 2); Table S4: Risk of bias of all included non-randomized clinical trials using the Risk of Bias in Non-randomized Studies of Interventions (ROBINS-I) tool; Table S5: Risk of bias of all included cohort studies using the Newcastle-Ottawa quality assessment scale; Table S6: Risk of bias of all included case-control studies using the Newcastle-Ottawa quality assessment scale; Table S7: Risk of bias of all included cross-sectional studies using the Agency for Healthcare Research and Quality scale
