# Supplementary material for: Immunogenicity and Safety of COVID-19 Vaccines among People Living with HIV: A Systematic Review and Meta-Analysis
Source: Vaccines (Basel). 2022 Sep 19;10(9):1569. doi: 10.3390/vaccines10091569 (PMC9503586; doi:10.3390/vaccines10091569)
Supplement: Supplementary file 1 [file vaccines-10-01569-s001.zip › vaccines-1899538-supplementary.pdf]

Supplemental Materials

Text S1: Detailed Search Strategies
Figure S1: Flowchart of study selection
Figure S2: The SMD of geometric mean titer among people living with HIV and healthy controls.
Figure S3: The incidence rates of adverse events among people living with HIV.
Figure S4: Sensitivity analysis of seroconversion rates by excluding studies with a high risk of bias.
Figure S5: Sensitivity analysis of risk ratio of seroconversion by excluding studies with a high risk of bias.
Figure S6: Sensitivity analysis of incidence rates of adverse events by excluding studies with a high risk of bias.
Figure S7: Sensitivity analysis of seroconversion rates by excluding studies with the number of people living with HIV less than 100.
Figure S8: Sensitivity analysis of risk ratio of seroconversion by excluding studies with the number of people living with HIV less than 100.
Figure S9 Sensitivity analysis of incidence rates of adverse events by excluding studies with the number of people living with HIV less than 100.
Figure S10: The publication bias of studies on seroconversion rates among people living with HIV after a first or second dose of COVID-19 vaccine
Figure S11: The publication bias of studies on seroconversion among people living with HIV compared with healthy controls after a first or second dose of COVID-19 vaccine
Figure S12: The publication bias of studies on incidence rates of adverse events among people living with HIV after a first or second dose of COVID-19 vaccine
Table S1: Characteristics and basic information of the studies included in the systematic review and meta-analysis for COVID-19 vaccine immunogenicity
Table S2: Characteristics and basic information of the studies included in the systematic review and meta-analysis for COVID-19 vaccine safety
Table S3: Risk of bias of all included randomized clinical trials using the revised Cochrane risk-of-bias tool for randomized trials (RoB 2)
Table S4: Risk of bias of all included non-randomized clinical trials using the Risk of Bias in Non-randomized Studies of Interventions (ROBINS-I) tool
Table S5: Risk of bias of all included cohort studies using the Newcastle-Ottawa quality assessment scale
Table S6: Risk of bias of all included case-control studies using the Newcastle-Ottawa quality assessment scale
Table S7: Risk of bias of all included cross-sectional studies using the Agency for Healthcare Research and Quality scale

Text S1: Detailed Search Strategies

Database: PubMed

Search terms: ((COVID-19[MeSH Terms]) OR (SARS-CoV-2[MeSH Terms]) OR (Severe Acute Respiratory Syndrome Coronavirus 2) OR (coronavirus disease 2019) OR (SARS-CoV*) OR (SARS Coronavirus*) OR (covid*)) AND ((HIV[MeSH Terms]) OR (Acquired Immunodeficiency Syndrome[MeSH Terms]) OR (HIV*) OR (Human immunodeficiency virus*) OR (Human immune deficiency virus*) OR (AIDS*) OR (Acquired Immunodeficiency Syndrome*) OR (acquired immune deficiency syndrome*)) AND ((COVID-19 Vaccines[MeSH Terms]) OR (Vaccines[MeSH Terms]) OR (Vaccination[MeSH Terms]) OR (Vaccin*)) AND (("2020/01/01"[Date - Publication] : "2022/04/29"[Date - Publication]))

Database: EMBASE

Search terms: ('severe acute respiratory syndrome coronavirus 2'/exp OR 'coronavirus disease 2019'/exp OR ('severe acute respiratory syndrome coronavirus 2' OR 'coronavirus disease 2019' OR 'sars-cov*' OR 'sars coronavirus*' OR 'covid*')) AND ('human immunodeficiency virus'/exp OR 'human immunodeficiency virus infection'/exp OR 'acquired immune deficiency syndrome'/exp OR ('hiv*' OR 'human immunodeficiency virus*' OR 'acquired immunodeficiency syndrome*' OR 'aids*' OR 'human immune deficiency virus*' OR 'acquired immune deficiency syndrome*')) AND ('sars-cov-2 vaccine'/exp OR 'vaccine'/exp OR 'vaccin*') AND [embase]/lim AND [01-01-2020]/sd NOT [30-04-2022]/sd

Database: Web of Science

Search terms: TS= (covid-19 OR Severe Acute Respiratory Syndrome Coronavirus 2 OR coronavirus disease 2019 OR SARS-CoV* OR SARS Coronavirus* OR covid*) AND TS= (HIV* OR Human immunodeficiency virus* OR Human immune deficiency virus* OR AIDS* OR acquired immunodeficiency syndrome* OR acquired immune deficiency syndrome*) AND TS= (Vaccin* OR COVID-19 Vaccin* OR SARS-CoV-2 vaccin*) AND ("2020/01/01"[Date - Publication]: "2022/04/29"[Date - Publication])

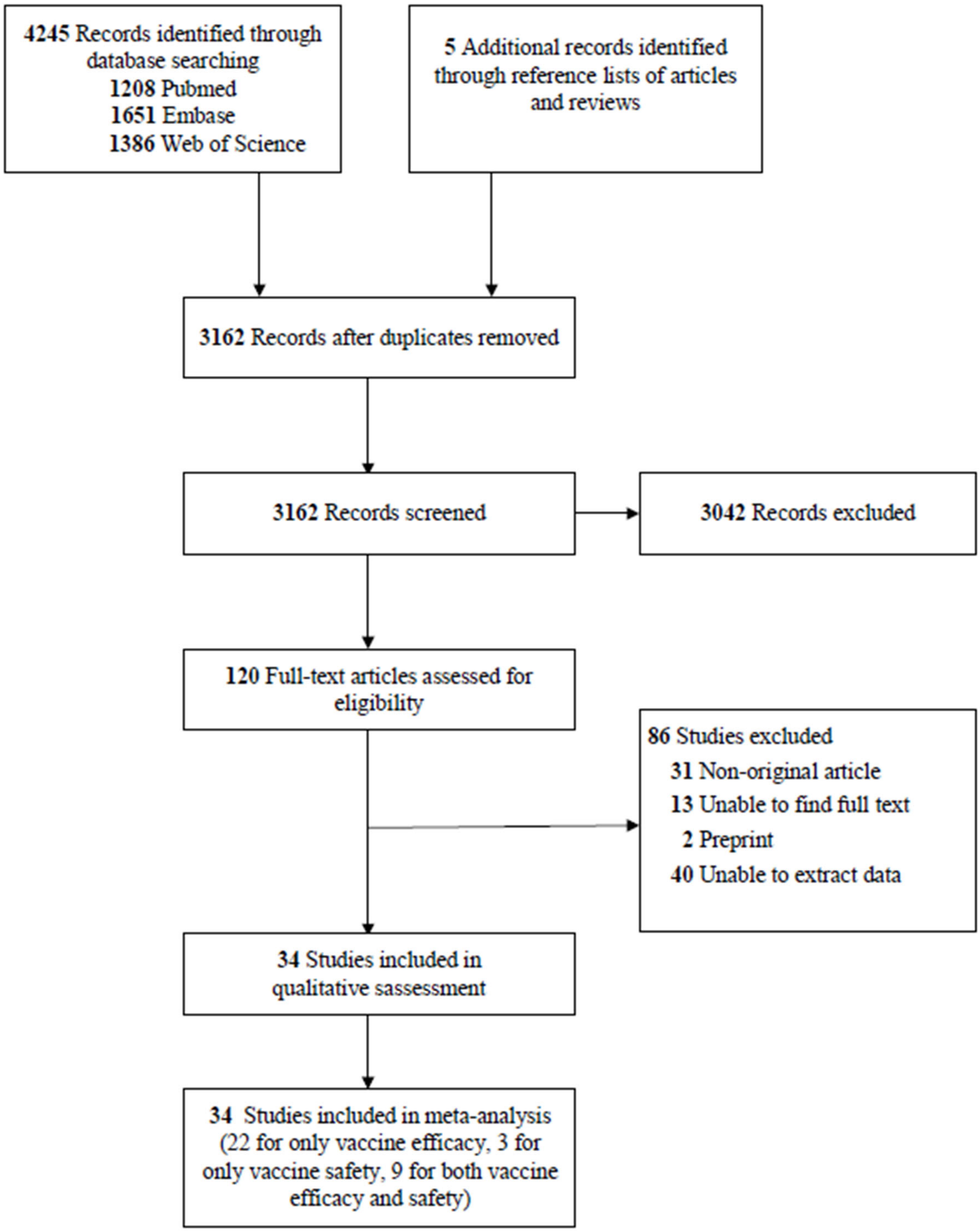

Figure S1: Flowchart of study selection

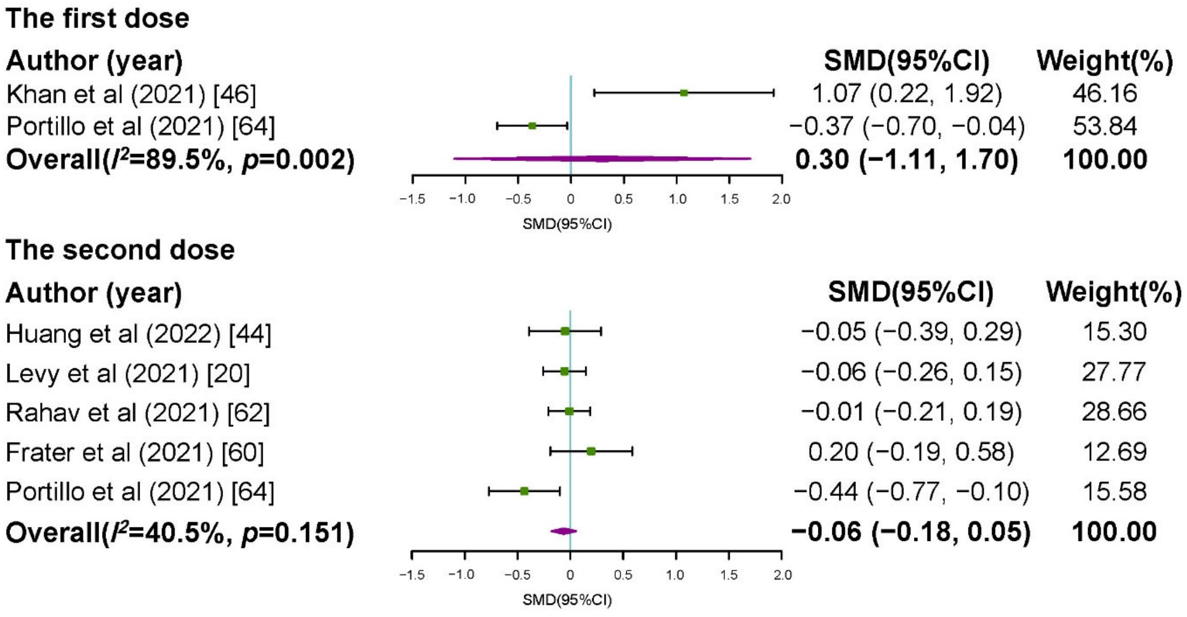

Figure S2: The SMD of geometric mean titer among people living with HIV and healthy controls.

SMD: standardized mean difference; CI: confidence interval

Total adverse events

Author (year)	No of PLWH	No of PLWH having adverse events	Incidence rate(%)(95%CI)	Weight(%)
**The first dose**				
Levy et al (2021) [20]	133	64	48.12 (39.38−56.95)	19.67
Portillo et al (2021) [64]	131	104	79.39 (71.45−85.96)	19.95
Netto et al (2022) [61]	169	60	35.50 (28.31−43.22)	19.90
Wu et al (2022) [57]	259	59	22.78 (17.82−28.38)	20.21
Milano et al (2022) [56]	440	208	47.27 (42.53−52.06)	20.27
**Overall(I^2=97.7%, p<0.001)**	**1132**	**495**	**46.55 (28.29−64.82)**	**100.00**
**The second dose**				
Bergman et al (2021) [59]	79	1	1.27 (0.03−6.85)	12.70
Ao et al (2022) [58]	139	18	12.95 (7.86−19.69)	12.57
Han et al (2022) [18]	47	9	19.15 (9.15−33.26)	12.11
Levy et al (2021) [20]	121	60	49.59 (40.37−58.82)	12.34
Portillo et al (2021) [64]	129	102	79.07 (71.03−85.73)	12.48
Netto et al (2022) [61]	189	55	29.10 (22.74−36.13)	12.52
Wu et al (2022) [57]	236	24	10.17 (6.63−14.75)	12.66
Milano et al (2022) [56]	440	207	47.05 (42.30−51.83)	12.62
**Overall(I^2=99.0%, p<0.001)**	**1380**	**476**	**30.96 (13.23−48.70)**	**100.00**

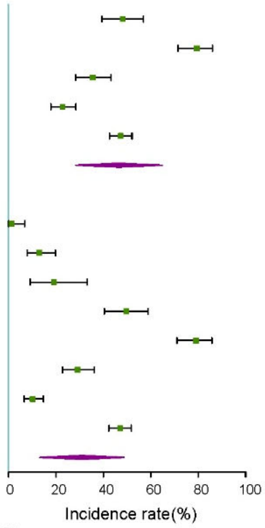

Systemic adverse events

Author (year)	No of PLWH	No of PLWH having adverse events	Incidence rate(%)(95%CI)	Weight(%)
**The first dose**				
Frater et al (2021) [60]	53	40	75.47 (61.72−86.24)	20.08
Levy et al (2021) [20]	133	26	19.55 (13.19−27.32)	20.85
Netto et al (2022) [61]	169	55	32.54 (25.55−40.17)	20.81
Speich et al (2022) [63]	342	21	6.14 (3.84−9.23)	21.21
Ruddy, Boyarsky et al (2021) [24]	14	10	71.43 (41.90−91.61)	17.04
**Overall(I^2=98.0%, p<0.001)**	**711**	**152**	**39.48 (17.58−61.38)**	**100.00**
**The second dose**				
Frater et al (2021) [60]	51	22	43.14 (29.35−57.75)	15.31
Levy et al (2021) [20]	121	60	49.59 (40.37−58.82)	17.74
Rahav et al (2021) [62]	143	28	19.58 (13.42−27.04)	18.79
Netto et al (2022) [61]	189	48	25.40 (19.36−32.22)	18.90
Speich et al (2022) [63]	339	63	18.58 (14.59−23.14)	19.57
Ruddy et al (2021) [24]	14	9	64.29 (35.14−87.24)	9.69
**Overall(I^2=91.3%, p<0.001)**	**857**	**230**	**33.75 (22.90−44.60)**	**100.00**

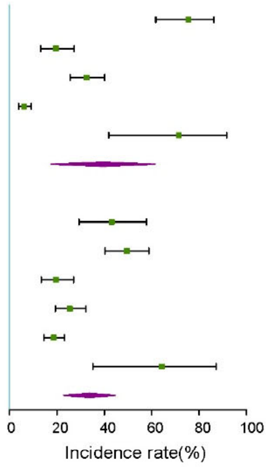

Local adverse events

Author (year)	No of PLWH	No of PLWH having adverse events	Incidence rate(%)(95%CI)	Weight(%)
**The first dose**				
Frater et al (2021) [60]	53	40	75.47 (61.72−86.24)	19.79
Levy et al (2021) [20]	133	54	40.60 (32.18−49.46)	20.33
Netto et al (2022) [61]	169	21	12.43 (7.86−18.37)	20.73
Speich et al (2022) [63]	342	25	7.31 (4.79−10.60)	20.89
Ruddy, et al (2021) [24]	14	12	85.71 (57.19−98.22)	18.25
**Overall(I^2=98.3%, p<0.001)**	**711**	**152**	**42.94 (21.14−64.74)**	**100.00**
**The second dose**				
Frater et al (2021) [60]	51	22	43.14 (29.35−57.75)	15.78
Levy et al (2021) [20]	121	31	25.62 (18.12−34.35)	16.90
Rahav et al (2021) [62]	143	58	40.56 (32.44−49.08)	16.86
Netto et al (2022) [61]	189	30	15.87 (10.97−21.88)	17.24
Speich et al (2022) [63]	339	31	9.14 (6.30−12.73)	17.42
Ruddy et al (2021) [24]	14	13	92.86 (66.13−99.82)	15.80
**Overall(I^2=97.5%, p<0.001)**	**857**	**185**	**36.98 (19.83−54.13)**	**100.00**

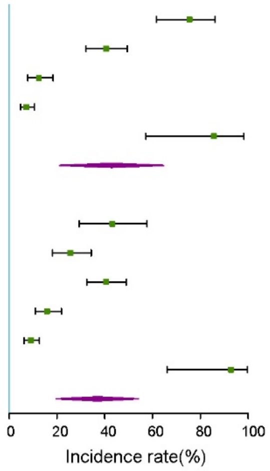

Figure S3: The incidence rates of adverse events among people living with HIV. PLWH: people living with HIV; CI: confidence interval.

The first dose

Author (year)	No of PLWH	No of PLWH with seroconversion	Seroconversion rate(%)(95%CI)	Weight(%)
Feng et al (2022) [40]	42	10	23.81 (12.05–39.45)	10.97
Heftdal et al (2022) [43]	269	218	81.04 (75.84–85.55)	11.35
Huang et al (2022) [44]	35	11	31.43 (16.85–49.29)	10.79
Jedicke et al (2022) [45]	88	56	63.64 (52.69–73.63)	11.13
Madhi et al (2021) [23]	36	31	86.11 (70.50–95.33)	11.07
Nault et al (2022) [49]	106	100	94.34 (88.09–97.89)	11.35
Netto et al (2022) [61]	214	41	19.16 (14.11–25.08)	11.33
Oyaert et al (2022) [51]	27	23	85.19 (66.27–95.81)	10.93
Tuan et al (2022) [53]	78	45	57.69 (45.98–68.81)	11.08
**Overall(I^2=98.6%, p<0.001)**	**895**	**535**	**60.40 (38.64–82.15)**	**100.00**

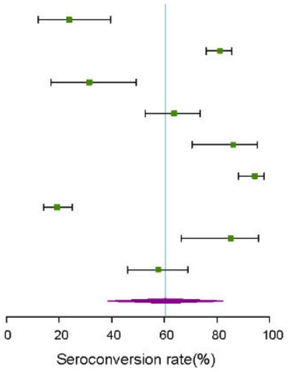

The second dose

Author (year)	No of PLWH	No of PLWH with seroconversion	Seroconversion rate(%)(95%CI)	Weight(%)
Antinori et al (2022) [37]	160	155	96.88 (92.86–98.98)	5.48
Ao et al (2022) [58]	139	121	87.05 (80.31–92.14)	2.85
Balcells et al (2022) [36]	55	39	70.91 (57.10–82.37)	0.87
Bergman et al (2021) [59]	79	78	98.73 (93.15–99.97)	5.74
Feng et al (2022) [40]	42	29	69.05 (52.91–82.38)	0.66
Gonzalez et al (2022) [22]	100	100	100.00 (96.38–100.00)	6.91
Haidar et al (2022) [41]	94	75	79.79 (70.25–87.37)	1.68
Han et al (2022) [18]	47	47	100.00 (92.45–100.00)	5.28
Hassold et al (2022) [42]	105	99	94.29 (87.98–97.87)	3.70
Heftdal et al (2022) [43]	269	269	100.00 (98.64–100.00)	7.49
Huang et al (2022) [44]	94	87	92.55 (85.26–96.95)	3.04
Jedicke et al (2022) [45]	52	50	96.15 (86.79–99.53)	3.09
Levy et al (2021) [20]	143	139	97.20 (92.99–99.23)	5.47
Liu et al (2021) [47]	55	55	100.00 (93.51–100.00)	5.74
Lv et al (2022) [48]	24	19	79.17 (57.85–92.87)	0.50
Madhi et al (2021) [23]	32	30	93.75 (79.19–99.23)	1.60
Netto et al (2022) [61]	204	185	90.69 (85.84–94.30)	4.11
Noe et al (2021) [21]	665	647	97.29 (95.76–98.39)	7.03
Ogbe et al (2022) [50]	42	35	83.33 (68.64–93.03)	0.98
Oyaert et al (2022) [51]	23	23	100.00 (85.18–100.00)	2.77
Rahav et al (2021) [62]	156	154	98.72 (95.45–99.84)	6.52
Speich et al (2022) [63]	341	341	100.00 (98.92–100.00)	7.53
Spinelli et al (2021) [52]	100	88	88.00 (79.98–93.64)	2.40
Tuan et al (2022) [53]	40	39	97.50 (86.84–99.94)	3.38
Wong et al (2022) [55]	213	202	94.84 (90.95–97.39)	5.17
**Overall(I^2=86.8%, p<0.001)**	**3274**	**3106**	**96.21 (95.02–97.41)**	**100.00**

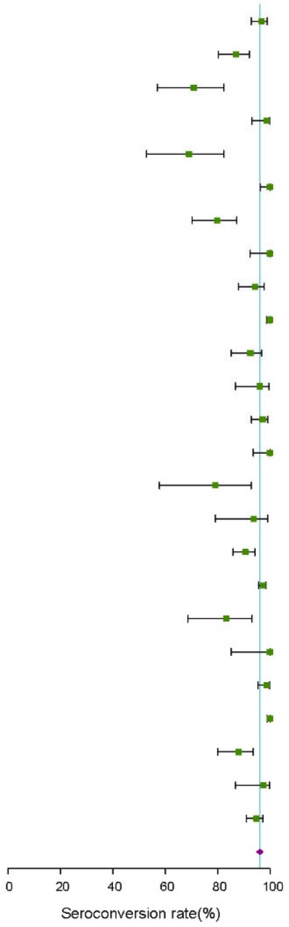

Figure S4: Sensitivity analysis of seroconversion rates by excluding studies with a high risk of bias.

PLWH: people living with HIV; CI: confidence interval.

The first dose

Author (year)	PLWH(n_s/N)	Healthy controls(n_s/N)	RR(95%CI)	Weight(%)
Feng et al (2022) [40]	10/42	20/28	0.33 (0.18−0.60)	8.59
Heftdal et al (2022) [43]	218/269	355/538	1.23 (1.13−1.34)	16.40
Jedicke et al (2022) [45]	56/88	41/41	0.64 (0.55−0.75)	15.66
Madhi et al (2021) [23]	31/36	18/23	1.10 (0.86−1.42)	14.25
Nault et al (2022) [49]	100/106	19/20	0.99 (0.89−1.11)	16.17
Netto et al (2022) [61]	41/214	114/295	0.50 (0.36−0.68)	13.24
Oyaert et al (2022) [51]	23/27	54/54	0.85 (0.73−1.00)	15.70
**Overall(I^2=93.7%, p<0.001)**	**479/782**	**621/999**	**0.79 (0.62−1.01)**	**100.00**

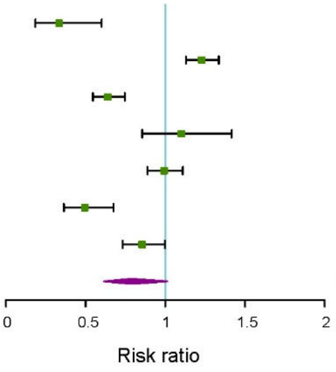

The second dose

Author (year)	PLWH(n_s/N)	Healthy controls(n_s/N)	RR(95%CI)	Weight(%)
Antinori et al (2022) [37]	155/160	168/168	0.97 (0.94−1.00)	7.96
Ao et al (2022) [58]	121/139	119/120	0.88 (0.82−0.94)	6.91
Balcells et al (2022) [38]	39/55	60/65	0.77 (0.64−0.92)	3.34
Bergman et al (2021) [59]	78/79	78/78	0.99 (0.96−1.01)	8.01
Feng et al (2022) [40]	29/42	20/28	0.97 (0.71−1.32)	1.59
Haidar et al (2022) [41]	75/94	159/172	0.86 (0.77−0.96)	5.38
Han et al (2022) [18]	47/47	18/18	1.00 (0.92−1.08)	6.39
Heftdal et al (2022) [43]	269/269	536/538	1.00 (1.00−1.01)	8.22
Jedicke et al (2022) [45]	50/52	41/41	0.96 (0.91−1.02)	7.31
Levy et al (2021) [20]	139/143	258/261	0.98 (0.95−1.01)	7.90
Lv et al (2022) [48]	19/24	21/24	0.90 (0.70−1.17)	2.15
Madhi et al (2021) [23]	30/32	22/23	0.98 (0.87−1.11)	4.90
Netto et al (2022) [61]	185/204	265/274	0.94 (0.89−0.98)	7.45
Oyaert et al (2022) [51]	23/23	52/52	1.00 (0.94−1.07)	6.94
Rahav et al (2021) [62]	154/156	269/272	1.00 (0.98−1.02)	8.06
Wong et al (2022) [55]	202/213	78/80	0.97 (0.93−1.02)	7.50
**Overall(I^2=95.7%, p<0.001)**	**1615/1732**	**2164/2214**	**0.96 (0.92−1.00)**	**100.00**

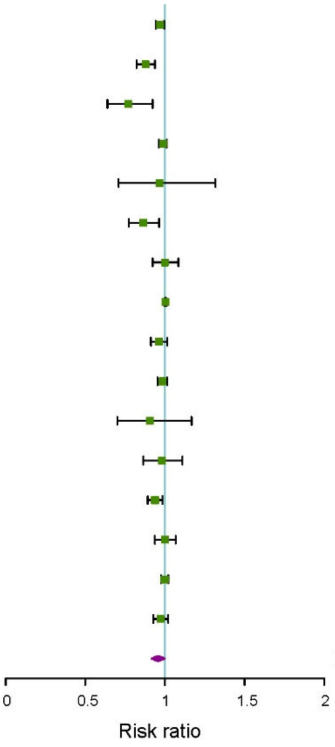

Figure S5: Sensitivity analysis of risk ratio of seroconversion by excluding studies with a high risk of bias.

PLWH: people living with HIV; n_s: number of people with seroconversion; N: group size; RR: risk ratio; CI: confidence interval.

Total adverse events

Author (year)	No of PLWH	No of PLWH having adverse events	Incidence rate(%)(95%CI)	Weight(%)
**The first dose**				
Levy et al (2021) [20]	133	64	48.12 (39.38–56.95)	19.67
Portillo et al (2021) [64]	131	104	79.39 (71.45–85.96)	19.95
Netto et al (2022) [61]	169	60	35.50 (28.31–43.22)	19.90
Wu et al (2022) [57]	259	59	22.78 (17.82–28.38)	20.21
Milano et al (2022) [56]	440	208	47.27 (42.53–52.06)	20.27
**Overall(I^2=97.7%, *p*<0.001)**	**1132**	**495**	**46.55 (28.29–64.82)**	**100.00**
**The second dose**				
Bergman et al (2021) [59]	79	1	1.27 (0.03–6.85)	12.70
Ao et al (2022) [58]	139	18	12.95 (7.86–19.69)	12.57
Han et al (2022) [18]	47	9	19.15 (9.15–33.26)	12.11
Levy et al (2021) [20]	121	60	49.59 (40.37–58.82)	12.34
Portillo et al (2021) [64]	129	102	79.07 (71.03–85.73)	12.48
Netto et al (2022) [61]	189	55	29.10 (22.74–36.13)	12.52
Wu et al (2022) [57]	236	24	10.17 (6.63–14.75)	12.66
Milano et al (2022) [56]	440	207	47.05 (42.30–51.83)	12.62
**Overall(I^2=99.0%, *p*<0.001)**	**1380**	**476**	**30.96 (13.23–48.70)**	**100.00**

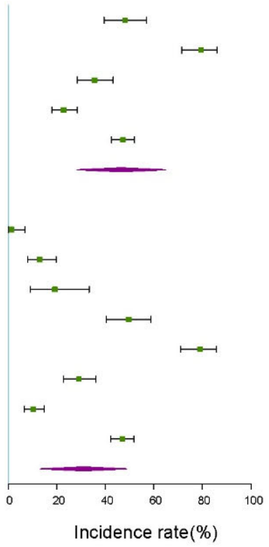

Syetemic adverse events

Author (year)	No of PLWH	No of PLWH having adverse events	Incidence rate(%)(95%CI)	Weight(%)
**The first dose**				
Levy et al (2021) [20]	133	26	19.55 (13.19–27.32)	26.92
Netto et al (2022) [61]	169	55	32.54 (25.55–40.17)	26.81
Speich et al (2022) [63]	342	21	6.14 (3.84–9.23)	27.90
Ruddy, Boyarsky et al (2021)[24]	14	10	71.43 (41.90–91.61)	18.37
**Overall(I^2=96.3%, *p*<0.001)**	**658**	**112**	**28.82 (11.57–46.08)**	**100.00**
**The second dose**				
Levy et al (2021) [20]	121	60	49.59 (40.37–58.82)	20.94
Rahav et al (2021) [62]	143	28	19.58 (13.42–27.04)	22.24
Netto et al (2022) [61]	189	48	25.40 (19.36–32.22)	22.38
Speich et al (2022) [63]	339	63	18.58 (14.59–23.14)	23.21
Ruddy, Boyarsky et al (2021)[24]	14	9	64.29 (35.14–87.24)	11.23
**Overall(I^2=92.0%, *p*<0.001)**	**806**	**208**	**31.95 (20.41–43.50)**	**100.00**

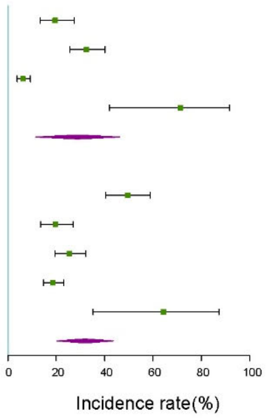

Local adverse events

Author (year)	No of PLWH	No of PLWH having adverse events	Incidence rate(%)(95%CI)	Weight(%)
**The first dose**				
Levy et al (2021) [20]	133	54	40.60 (32.18–49.46)	25.52
Netto et al (2022) [61]	169	21	12.43 (7.86–18.37)	26.38
Speich et al (2022) [63]	342	25	7.31 (4.79–10.60)	26.72
Ruddy, Boyarsky et al (2021)[24]	14	12	85.71 (57.19–98.22)	21.39
**Overall(I^2=97.5%, *p*<0.001)**	**658**	**112**	**33.92 (15.16–52.68)**	**100.00**
**The second dose**				
Levy et al (2021) [20]	121	31	25.62 (18.12–34.35)	20.07
Rahav et al (2021) [62]	143	58	40.56 (32.44–49.08)	20.02
Netto et al (2022) [61]	189	30	15.87 (10.97–21.88)	20.46
Speich et al (2022) [63]	339	31	9.14 (6.30–12.73)	20.68
Ruddy, Boyarsky et al (2021)[24]	14	13	92.86 (66.13–99.82)	18.76
**Overall(I^2=97.8%, *p*<0.001)**	**806**	**163**	**35.82 (17.14–54.50)**	**100.00**

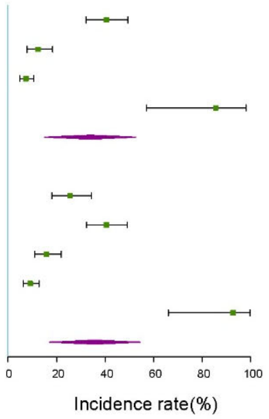

Figure S6: Sensitivity analysis of incidence rates of adverse events by excluding studies with a high risk of bias.

PLWH: people living with HIV; CI: confidence interval.

The first dose

Author (year)	No of PLWH	No of PLWH with seroconversion	Seroconversion rate(%)(95% CI)	Weight(%)
Heftdal et al (2022) [43]	269	218	81.04 (75.84−85.55)	33.34
Nault et al (2022) [49]	106	100	94.34 (88.09−97.89)	33.36
Netto et al (2022) [61]	214	41	19.16 (14.11−25.08)	33.31
**Overall(I^2=99.6%, p<0.001)**	**589**	**359**	**64.87 (21.49−100.00)**	**100.00**

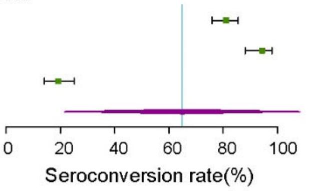

The second dose

Author (year)	No of PLWH	No of PLWH with seroconversion	Seroconversion rate(%)(95% CI)	Weight(%)
Antinori et al (2022) [37]	160	155	96.88 (92.86−98.98)	8.06
Ao et al (2022) [58]	139	121	87.05 (80.31−92.14)	3.49
Gonzalez et al (2022) [22]	100	100	100.00 (96.38−100.00)	11.43
Hassold et al (2022) [42]	105	99	94.29 (87.98−97.87)	4.79
Heftdal et al (2022) [43]	269	269	100.00 (98.64−100.00)	13.07
Levy et al (2021) [20]	143	139	97.20 (92.99−99.23)	8.04
Netto et al (2022) [61]	204	185	90.69 (85.84−94.30)	5.47
Noe et al (2021) [21]	665	647	97.29 (95.76−98.39)	11.75
Rahav et al (2021) [62]	156	154	98.72 (95.45−99.84)	10.43
Speich et al (2022) [63]	341	341	100.00 (98.92−100.00)	13.18
Spinelli et al (2021) [52]	100	88	88.00 (79.98−93.64)	2.85
Wong et al (2022) [55]	213	202	94.84 (90.95−97.39)	7.42
**Overall(I^2=88.4%, p<0.001)**	**2595**	**2500**	**97.11 (95.90−98.32)**	**100.00**

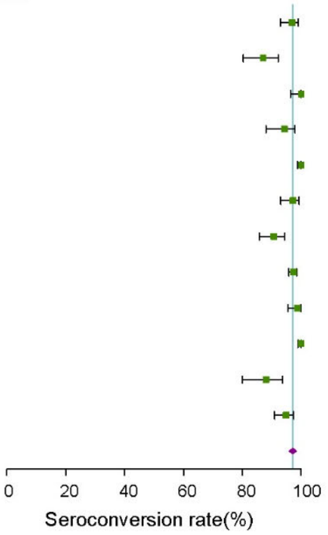

Figure S7: Sensitivity analysis of seroconversion rates by excluding studies with number of people living with HIV less than 100.

PLWH: people living with HIV; CI: confidence interval.

The first dose

Author (year)	PLWH(n_s/N)	Healthy controls(n_s/N)	RR(95%CI)	Weight(%)
Heftdal et al (2022) [43]	218/269	355/538	1.23 (1.13−1.34)	36.08
Nault et al (2022) [49]	100/106	19/20	0.99 (0.89−1.11)	35.51
Netto et al (2022) [61]	41/214	114/295	0.50 (0.36−0.68)	28.41
**Overall(I^2=95.1%, p<0.001)**	**359/589**	**488/853**	**0.88 (0.62−1.24)**	**100.00**

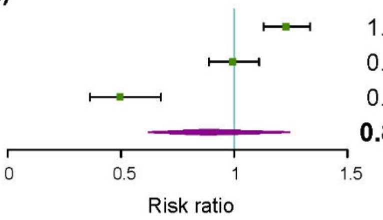

The second dose

Author (year)	PLWH(n_s/N)	Healthy controls(n_s/N)	RR(95%CI)	Weight(%)
Antinori et al (2022) [37]	155/160	168/168	0.97 (0.94−1.00)	14.87
Ao et al (2022) [58]	121/139	119/120	0.88 (0.82−0.94)	12.38
Heftdal et al (2022) [43]	269/269	536/538	1.00 (1.00−1.01)	15.51
Levy et al (2021) [20]	139/143	258/261	0.98 (0.95−1.01)	14.73
Netto et al (2022) [61]	185/204	265/274	0.94 (0.89−0.98)	13.63
Rahav et al (2021) [62]	154/156	269/272	1.00 (0.98−1.02)	15.12
Wong et al (2022) [55]	202/213	78/80	0.97 (0.93−1.02)	13.76
**Overall(I^2=96.7%, p<0.001)**	**1225/1284**	**1693/1713**	**0.97 (0.92−1.02)**	**100.00**

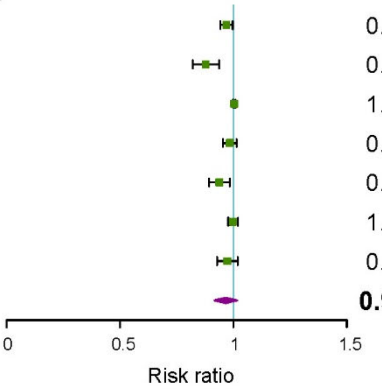

Figure S8: Sensitivity analysis of risk ratio of seroconversion by excluding studies with number of people living with HIV less than 100.

PLWH: people living with HIV; n_s: number of people with seroconversion; N: group size; RR: risk ratio; CI: confidence interval

Total adverse events

Author (year)	No of PLWH	No of PLWH having adverse events	Incidence rate(%)(95%CI)	Weight(%)
**The first dose**				
Levy et al (2021) [20]	133	64	48.12 (39.38–56.95)	19.67
Portillo et al (2021) [64]	131	104	79.39 (71.45–85.96)	19.95
Netto et al (2022) [61]	169	60	35.50 (28.31–43.22)	19.90
Wu et al (2022) [57]	259	59	22.78 (17.82–28.38)	20.21
Milano et al (2022) [56]	440	208	47.27 (42.53–52.06)	20.27
**Overall(I^2=97.7%, p<0.001)**	**1132**	**495**	**46.55 (28.29–64.82)**	**100.00**
**The second dose**				
Ao et al (2022) [58]	139	18	12.95 (7.86–19.69)	16.72
Levy et al (2021) [20]	121	60	49.59 (40.37–58.82)	16.41
Portillo et al (2021) [64]	129	102	79.07 (71.03–85.73)	16.60
Netto et al (2022) [61]	189	55	29.10 (22.74–36.13)	16.65
Wu et al (2022) [57]	236	24	10.17 (6.63–14.75)	16.83
Milano et al (2022) [56]	440	207	47.05 (42.30–51.83)	16.79
**Overall(I^2=98.7%, p<0.001)**	**1254**	**466**	**37.88 (17.47–58.29)**	**100.00**

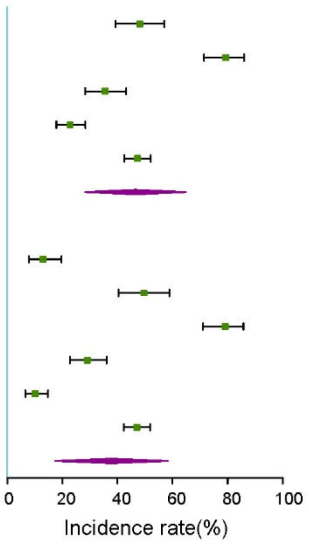

Systemic adverse events

Author (year)	No of PLWH	No of PLWH having adverse events	Incidence rate(%)(95%CI)	Weight(%)
**The first dose**				
Levy et al (2021) [20]	133	26	19.55 (13.19–27.32)	32.84
Netto et al (2022) [61]	169	55	32.54 (25.55–40.17)	32.66
Speich et al (2022) [63]	342	21	6.14 (3.84–9.23)	34.51
**Overall(I^2=96.4%, p<0.001)**	**644**	**102**	**19.17 (2.91–35.42)**	**100.00**
**The second dose**				
Levy et al (2021) [20]	121	60	49.59 (40.37–58.82)	23.33
Rahav et al (2021) [62]	143	28	19.58 (13.42–27.04)	25.05
Netto et al (2022) [61]	189	48	25.40 (19.36–32.22)	25.24
Speich et al (2022) [63]	339	63	18.58 (14.59–23.14)	26.37
**Overall(I^2=92.5%, p<0.001)**	**792**	**199**	**27.79 (16.57–39.00)**	**100.00**

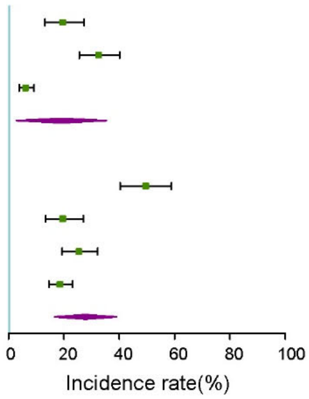

Local adverse events

Author (year)	No of PLWH	No of PLWH having adverse events	Incidence rate(%)(95%CI)	Weight(%)
**The first dose**				
Levy et al (2021) [20]	133	54	40.60 (32.18–49.46)	31.61
Netto et al (2022) [61]	169	21	12.43 (7.86–18.37)	33.75
Speich et al (2022) [63]	342	25	7.31 (4.79–10.60)	34.64
**Overall(I^2=96.4%, p<0.001)**	**644**	**100**	**19.56 (4.58–34.54)**	**100.00**
**The second dose**				
Levy et al (2021) [20]	121	31	25.62 (18.12–34.35)	24.17
Rahav et al (2021) [62]	143	58	40.56 (32.44–49.08)	24.01
Netto et al (2022) [61]	189	30	15.87 (10.97–21.88)	25.52
Speich et al (2022) [63]	339	31	9.14 (6.30–12.73)	26.30
**Overall(I^2=95.0%, p<0.001)**	**792**	**150**	**22.39 (10.04–34.74)**	**100.00**

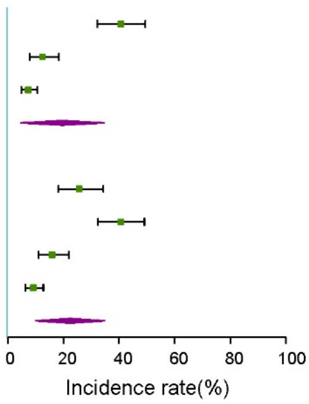

Figure S9 Sensitivity analysis of incidence rates of adverse events by excluding studies with number of people living with HIV less than 100.
PLWH: people living with HIV; CI: confidence interval.

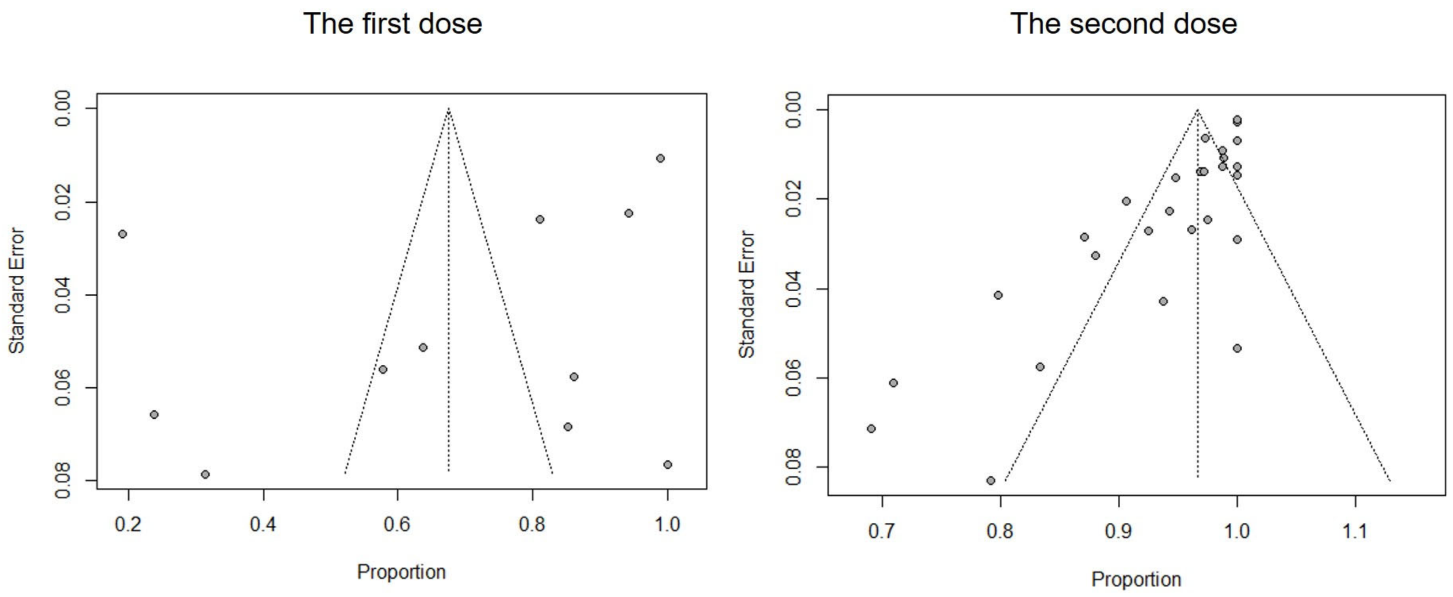

Figure S10: The publication bias of studies on seroconversion rates among people living with HIV after a first or second dose of COVID-19 vaccine

Egger's test:

The first dose: Test result: t = -1.83, df = 9, p-value = 0.1007

The second dose: Test result: t = -6.51, df = 26, p-value < 0.0001

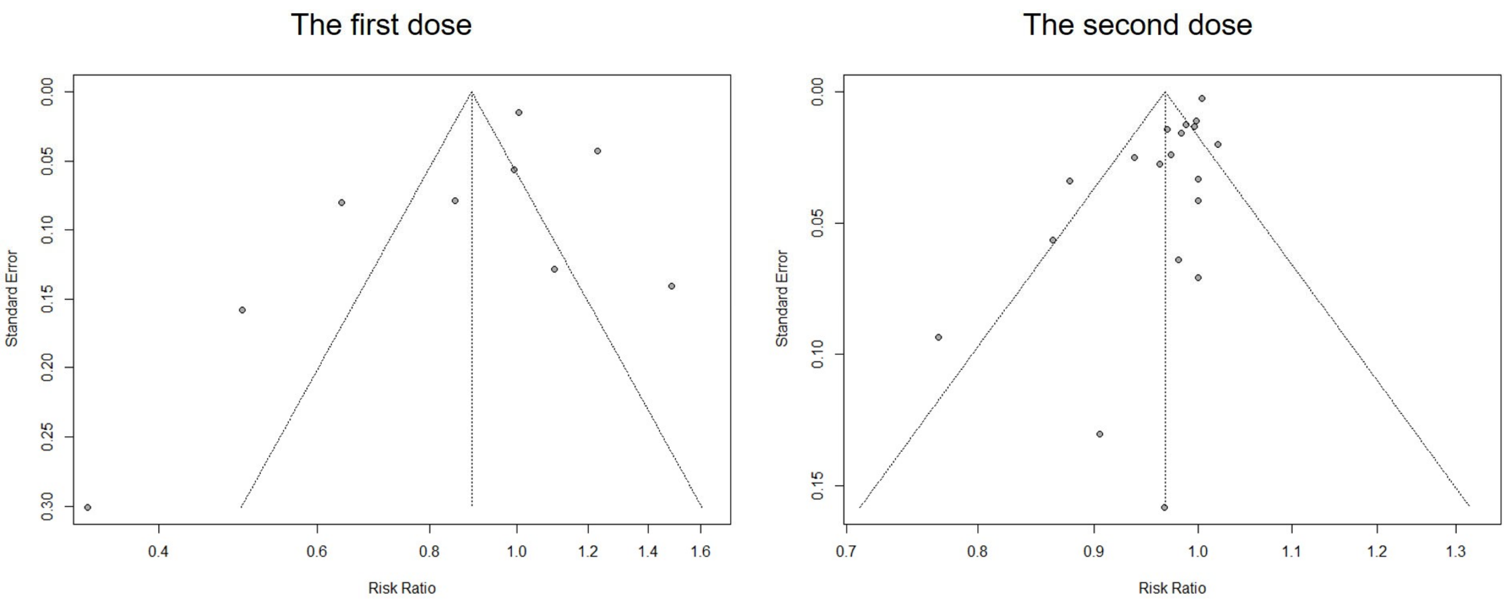

Figure S11: The publication bias of studies on seroconversion among people living with HIV compared with healthy controls after a first or second dose of COVID-19 vaccine

Egger's test:

The first dose Egger's test: Test result: t = -1.00, df = 7, p-value = 0.3502

The second dose Egger's test: Test result: t = -3.81, df = 17, p-value = 0.0014

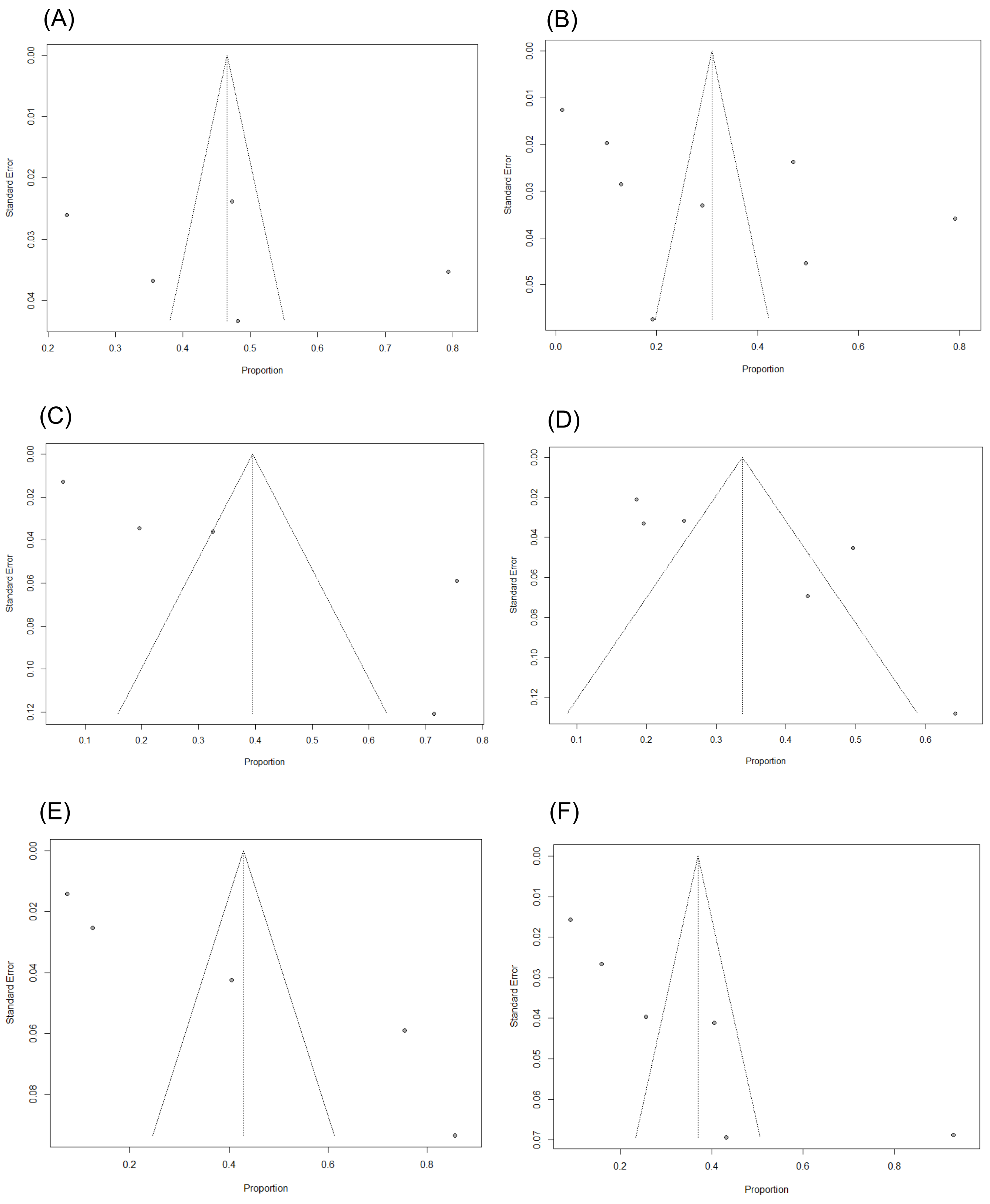

Figure S12: The publication bias of studies on incidence rates of adverse events among people living with HIV after a first or second dose of COVID-19 vaccine: (A) Total adverse events after the first dose; (B) Total adverse events after the second dose; (C) System adverse events after the first dose; (D) System adverse events after the second dose; (E) Local adverse events after the first dose; (F) Local adverse events after the second dose.

Table S1: Characteristics and basic information of the studies included in the systematic review and meta-analysis for COVID-19 vaccine immunogenicity

**Author**	**Publication year**	**Study design**	**Country**	**Risk of bias**	**Vaccine type**	**Vaccine dose**	**Time after vaccination**	**CD4 cell count**	**No of PLWH**	**No of PLWH with seroconversion**	**No of healthy controls**	**No of healthy controls with seroconversion**
Antinori et al	2022	cohort study	Italy	Moderate	BNT162b2 or mRNA-1273	2	after 30d		160	155	168	168
Antinori et al	2022	cohort study	Italy	Moderate	BNT162b2 or mRNA-1273	2	after 30d	<200	30	26	168	168
Antinori et al	2022	cohort study	Italy	Moderate	BNT162b2 or mRNA-1273	2	after 30d	200-500	53	53	168	168
Antinori et al	2022	cohort study	Italy	Moderate	BNT162b2 or mRNA-1273	2	after 30d	>500	77	76	168	168
Ao et al	2022	cohort study	China	Moderate	BBIBP-CorV, Corona Vac	2	≥21d		139	121	120	119
Balcells et al	2022	cohort study	Chile	Low	CoronaVac	2	≥56d		55	39	65	60
Bergman et al	2021	non-randomized clinical trials	Sweden	Moderate	BNT162b2	2	after 14d		79	78	78	78
Bergman et al	2021	non-randomized clinical trials	Sweden	Moderate	BNT162b2	2	after 14d	>300	54	54	78	78
Bergman et al	2021	non-randomized clinical trials	Sweden	Moderate	BNT162b2	2	after 14d	≤300	25	24	78	78
Brumme et al	2022	cohort study	Canada	High	BNT162b2 or mRNA-1273 or ChAdOx1	1	after 30d		92	91	137	135
Brumme et al	2022	cohort study	Canada	High	BNT162b2 or mRNA-1273 or ChAdOx1-S	2	after 30d		92	91	137	136
Feng et al	2022	non-randomized clinical trials	China	Moderate	BBIBP-CorV	1	after 28d		42	10	28	20
Feng et al	2022	non-randomized clinical trials	China	Moderate	BBIBP-CorV	2	after 28d		42	29	28	20
Frater et al	2021	non-randomized clinical trials	UK	High	ChAdOx1	2	after 14d		54	54	50	49
Gonzalez et al	2022	cross-sectional study	Spain	Low	mRNA-1273 and BNT162b2	2	after 28d		100	100		
Haidar et al	2022	cohort study	US	Low	any vaccine	2	≥14d		94	75	172	159
Han et al	2022	cohort study	China	Low	Sinovac CoronaVac or Sinopharm	2	after 14d		47	47	18	18

Hassold et al	2022	cross-sectional study	France	Low	BNT162b2 or mRNA-1273 or ChAdOx1-S	2	after 8-150d		105	99		
Hassold et al	2022	cross-sectional study	France	Low	BNT162b2 or mRNA-1273 or ChAdOx1-S	2	after 8-150d	<500	54	48		
Hassold et al	2022	cross-sectional study	France	Low	BNT162b2 or mRNA-1273 or ChAdOx1-S	2	after 8-150d	>500	51	51		
Heftdal et al	2022	cohort study	Denmark	Moderate	BNT162b2	1	after 14d		269	218	538	355
Heftdal et al	2022	cohort study	Denmark	Moderate	BNT162b2	2	after 7d		269	269	538	536
Huang et al	2022	cross-sectional study	China	Low	Sinovac CoronaVac or Sinopharm	1			35	11		
Huang et al	2022	cross-sectional study	China	Low	Sinovac CoronaVac or Sinopharm	2			94	87		
Jedicke et al	2022	cohort study	Germany	Moderate	BNT162b2	1	after 18.7d		88	56	41	41
Jedicke et al	2022	cohort study	Germany	Moderate	BNT162b2	2	after 35d		52	50	41	41
Khan et al	2021	non-randomized clinical trials	South Africa	High	Ad26.CoV2.S	1	after 74d		8	8	24	16
Levy et al	2021	cohort study	Israel	Moderate	BNT162b2	2	after 18d		143	139	261	258
Liu et al	2021	cross-sectional study	China	Low	CoronaVac or BBIBP-CorV	2	after 14d		55	55		
Lv et al	2022	non-randomized clinical trials	China	Low	CoronaVac or BBIBP-CorV	2	after 40d		24	19	24	21
Madhi et al	2021	RCT	South Africa	Moderate	ChAdOx1-S	1	after 28d		36	31	23	18
Madhi et al	2021	RCT	South Africa	Moderate	ChAdOx1-S	2	after 14d		32	30	23	22
Nault et al	2022	cohort study	Canada	Moderate	mRNA-1273 or BNT162b2	1	after 21-28d		106	100	20	19
Netto et al	2022	cohort study	Brazil	Low	CoronaVac	1	after 28d		214	41	295	114
Netto et al	2022	cohort study	Brazil	Low	CoronaVac	2	after 42d		204	185	274	265
Netto et al	2022	cohort study	Brazil	Low	CoronaVac	1	after 28d	<500	64	10	295	114
Netto et al	2022	cohort study	Brazil	Low	CoronaVac	1	after 28d	≥500	150	31	295	114
Netto et al	2022	cohort study	Brazil	Low	CoronaVac	2	after 42d	<500	62	51	274	265
Netto et al	2022	cohort study	Brazil	Low	CoronaVac	2	after 42d	≥500	142	134	274	265

Noe et al	2021	cross-sectional study	Germany	Moderate	BNT162b2 or mRNA-1273 or ChAdOx1-S or COVID-19 Vaccine Janssen	2			665	647		
Portillo et al	2021	cohort study	Swiss	Moderate	mRNA1273 or BNT162b2	1	after 28d		129		49	
Ogbe et al	2022	non-randomized clinical trials	UK	Moderate	ChAdOx1-S	2	after 180d		42	35		
Oyaert et al	2022	cohort study	Belgium	Low	BNT162b2	1	after 21-28d	<350	27	23	54	54
Oyaert et al	2022	cohort study	Belgium	Low	BNT162b2	2	after 10-14d	<350	23	23	52	52
Rahav et al	2021	cohort study	Israel	Moderate	BNT162b2	2	after 19d		156	154	272	269
Speich et al	2022	RCT	Swiss	Moderate	mRNA-1273 or BNT162b2	2	after 56d		341	341		
Spinelli et al	2021	case-control study	US	Low	mRNA-1273 or BNT162b2	2	after 35d		100	88		
Tuan et al	2022	cross-sectional study	US	Low	BNT162b2	1	after 21d		78	45		
Tuan et al	2022	cross-sectional study	US	Low	BNT162b2	2	after 14-21d		40	39		
Woldemeskel et al	2022	cohort study	US	High	BNT162b2	2	after 7-17d		12	12	17	17
Wong et al	2022	cohort study	China	Low	BNT162b2 or CoronaVac	2	after 14-42d		213	202	80	78
Wong et al	2022	cohort study	China	Low	CoronaVac	2	after 14-42d		74	64	32	30
Wong et al	2022	cohort study	China	Low	BNT162b2	2	after 14-42d		139	138	48	48

Table S2: Characteristics and basic information of the studies included in the systematic review and meta-analysis for COVID-19 vaccine safety

**Author**	**Publication year**	**Study design**	**Country**	**Risk of bias**	**Vaccine type**	**Vaccine dose**	**Type of adverse events**	**No of PLWH**	**No of PLWH having adverse events**	**No of healthy controls**	**No of healthy controls having adverse events**
Ao et al	2022	cohort study	China	Moderate	BBIBP-CorV, Corona Vac	2	total	139	18	120	16
Bergman et al	2021	non-randomized clinical trials	Sweden	Moderate	BNT162b2	2	total	79	1	78	0
Frater et al	2021	non-randomized clinical trials	UK	High	ChAdOx1-S	1	system	53	40	50	43
Frater et al	2021	non-randomized clinical trials	UK	High	ChAdOx1-S	1	local	53	40	50	44
Frater et al	2021	non-randomized clinical trials	UK	High	ChAdOx1-S	2	system	51	22	49	32
Frater et al	2021	non-randomized clinical trials	UK	High	ChAdOx1-S	2	local	51	22	49	37
Han et al	2022	cohort study	China	Low	Sinovac CoronaVac or Sinopharm	2	total	47	9		
Levy et al	2021	cohort study	Israel	Moderate	BNT162b2	2	total	121	60		
Levy et al	2021	cohort study	Israel	Moderate	BNT162b2	1	total	133	64		
Levy et al	2021	cohort study	Israel	Moderate	BNT162b2	1	system	133	26		
Levy et al	2021	cohort study	Israel	Moderate	BNT162b2	1	local	133	54		
Levy et al	2021	cohort study	Israel	Moderate	BNT162b2	2	system	121	60		
Levy et al	2021	cohort study	Israel	Moderate	BNT162b2	2	local	121	31		
Milano et al	2022	cohort study	Italy	Moderate	BNT162b2	2	total	440	207		
Milano et al	2022	cohort study	Italy	Moderate	BNT162b2	1	total	440	208		
Netto et al	2022	cohort study	Brazil	Low	CoronaVac	2	total	189	55	265	91
Netto et al	2022	cohort study	Brazil	Low	CoronaVac	1	total	169	60	296	122
Netto et al	2022	cohort study	Brazil	Low	CoronaVac	2	system	189	48	265	78
Netto et al	2022	cohort study	Brazil	Low	CoronaVac	1	system	169	55	296	97
Netto et al	2022	cohort study	Brazil	Low	CoronaVac	1	local	169	21	296	61
Netto et al	2022	cohort study	Brazil	Low	CoronaVac	2	local	189	30	265	45

Portillo et al	2021	cohort study	Swiss	Moderate	mRNA1273 or BNT162b2	2	total	129	102		
Portillo et al	2021	cohort study	Swiss	Moderate	mRNA1273 or BNT162b2	1	total	131	104		
Rahav et al	2021	cohort study	Israel	Moderate	BNT162b2	2	system	143	28	272	57
Rahav et al	2021	cohort study	Israel	Moderate	BNT162b2	2	local	143	58	272	199
Ruddy et al	2021	cohort study	US	Moderate	BNT162b2 or mRNA-1273	2	system	14	9		
Ruddy et al	2021	cohort study	US	Moderate	BNT162b2 or mRNA-1273	1	system	14	10		
Ruddy et al	2021	cohort study	US	Moderate	BNT162b2 or mRNA-1273	1	local	14	12		
Ruddy et al	2021	cohort study	US	Moderate	BNT162b2 or mRNA-1273	2	local	14	13		
Speich et al	2022	RCT	Swiss	Moderate	mRNA-1273 or BNT162b2	2	system	339	63		
Speich et al	2022	RCT	Swiss	Moderate	mRNA-1273 or BNT162b2	1	system	342	21		
Speich et al	2022	RCT	Swiss	Moderate	mRNA-1273 or BNT162b2	2	local	339	31		
Speich et al	2022	RCT	Swiss	Moderate	mRNA-1273 or BNT162b2	1	local	342	25		
Wu et al	2022	cross-sectional study	China		Sinopharm	1	total	259	59		
Wu et al	2022	cross-sectional study	China		Sinopharm	2	total	236	24		

Table S3: Risk of bias of all included randomized clinical trials using the revised Cochrane risk-of-bias tool for randomized trials (RoB 2)

Author	Bias arising from the randomization process	Bias due to deviations from intended interventions	Bias due to missing outcome data	Bias in measurement of the outcome	Bias in selection of the reported result	Total risk
Madhi et al	low risk	low risk	low risk	some concerns	low risk	some concerns
Speich et al	some concerns	low risk	low risk	low risk	low risk	some concerns

Table S4: Risk of bias of all included non-randomized clinical trials using the Risk of Bias in Non-randomized Studies of Interventions (ROBINS-I) tool

Author	Bias due to confounding	Bias in selection of participants into the study	Bias in classification of interventions	Bias due to deviations from intended interventions	Bias due to missing data	Bias in measurement of outcomes	Bias in selection of the reported result	Overall bias
Bergman et al	moderate	low	low	low	low	low	low	moderate
Feng et al	moderate	low	low	low	low	low	low	moderate
Frater et al	serious	low	moderate	low	low	moderate	low	serious
Khan et al	serious	moderate	low	low	moderate	low	low	serious
Ogbe et al	moderate	low	moderate	low	low	low	moderate	moderate
Lv et al	low	low	low	low	low	low	low	low

Table S5: Risk of bias of all included cohort studies using the Newcastle-Ottawa quality assessment scale

Author	Selection				Comparability	Outcome			Total score	Risk level*
	Representativeness of the Exposed Cohort	Selection of the Non-Exposed Cohort	Ascertainment of Exposure	Demonstration That Outcome of Interest Was Not Present at Start of Study		Assessment of Outcome	Was Follow-Up Long Enough for Outcomes to Occur	Adequacy of Follow Up of Cohorts		
Antinori et al	0	0	1	1	2	1	0	1	6	moderate
Balcells et al	0	1	1	1	2	1	1	1	8	low
Ao et al	0	0	1	1	2	1	1	0	6	moderate
Haidar et al	1	1	0	1	1	1	1	1	7	low
Han et al	0	1	1	1	2	1	1	1	8	low
Heftdal et al	0	1	1	0	1	1	1	1	6	moderate
Jedicke et al	0	0	1	1	1	1	1	0	5	moderate
Levy et al	0	0	1	1	2	1	0	1	6	moderate
Rahav et al	0	0	1	1	1	1	1	1	6	moderate
Oyaert et al	0	1	1	1	1	1	1	1	7	low
Portillo et al	1	0	1	0	1	1	1	1	6	moderate
Netto et al	0	0	1	1	2	1	1	1	7	low
Wong et al	0	1	0	1	2	1	1	1	7	low
Woldemeskel et al	0	0	1	0	1	0	0	1	3	high
Nault et al	0	0	1	0	1	1	1	1	5	moderate
Brumme et al	0	0	1	0	1	0	1	0	3	high
Milanoc et al	0	0	1	1	0	1	1	1	5	moderate
Ruddy et al	0	1	0	0	2	1	1	1	6	moderate

*Low (total score ≥ 7), moderate (total score 5-6), and high (total score ≤ 4) risk of bias.

Table S6: Risk of bias of all included case-control studies using the Newcastle-Ottawa quality assessment scale

Author	Selection				Comparability	Exposure			Total score	Risk level*
	Is the case definition adequate?	Representativeness of the cases	Selection of Controls	Definition of Controls	Study controls for antibody positive rate, antibody (the most important factor)<br>Study controls for any additional factor	Ascertainment of exposure	Same method of ascertainment for cases and controls	Non-Response rate		
Spinelli et al	1	1	0	1	1	1	1	0	7	low

*Low (total score ≥ 7), moderate (total score 5-6), and high (total score ≤ 4) risk of bias.

Table S7: Risk of bias of all included cross-sectional studies using the Agency for Healthcare Research and Quality scale

Author	1) Define the source of information (survey, record review)	2) List inclusion and exclusion criteria for exposed and unexposed subjects (cases and controls) or refer to previous publications	3) Indicate time period used for identifying patients	4) Indicate whether or not subjects were consecutive if not population-based	5) Indicate if evaluators of subjective components of study were masked to other aspects of the status of the participants	6) Describe any assessments undertaken for quality assurance purposes (e.g., test/retest of primary outcome measurements)	7) Explain any patient exclusions from analysis	8) Describe how confounding was assessed and/or controlled.	9) If applicable, explain how missing data were handled in the analysis	10) Summarize patient response rates and completeness of data collection	11) Clarify what follow-up, if any, was expected and the percentage of patients for which incomplete data or follow-up was obtained	Total scores	Article quality*
Gonzalez et al	yes	yes	uncelar	yes	no	no	yes	yes	yes	yes	yes	8	high
Hassold et al	yes	yes	yes	yes	no	yes	no	yes	yes	yes	yes	9	high
Huang et al	yes	yes	yes	yes	no	yes	yes	yes	yes	yes	yes	10	high
Liu et al	yes	yes	yes	yes	no	no	yes	yes	yes	yes	yes	9	high
Noe et al	yes	yes	yes	no	no	no	yes	yes	unclear	yes	no	6	moderate
Wu et al	unclear	unclear	yes	yes	no	yes	yes	yes	yes	no	yes	7	moderate
Tuan et al	yes	yes	yes	yes	yes	yes	yes	yes	unclear	yes	yes	10	high

Note: low quality (high risk of bias) (0–3 score), moderate quality (moderate risk of bias) (4–7score), high quality (low risk of bias) (8–11score) of articles.
